# Hypoxia signaling in human health and diseases: implications and prospects for therapeutics

**DOI:** 10.1038/s41392-022-01080-1

**Published:** 2022-07-07

**Authors:** Zhen Luo, Mingfu Tian, Ge Yang, Qiaoru Tan, Yubing Chen, Geng Li, Qiwei Zhang, Yongkui Li, Pin Wan, Jianguo Wu

**Affiliations:** 1 Guangdong Provincial Key Laboratory of Virology, Institute of Medical Microbiology, Jinan University, Guangzhou, 510632 China; 2 Foshan Institute of Medical Microbiology, Foshan, 528315 China; 3 The First Affiliated Hospital of Jinan University, Guangzhou, 510632 China; 4 The Affiliated Shunde Hospital of Jinan University, Foshan, 528303 China

**Keywords:** Molecular medicine, Cell biology

## Abstract

Molecular oxygen (O_2_) is essential for most biological reactions in mammalian cells. When the intracellular oxygen content decreases, it is called hypoxia. The process of hypoxia is linked to several biological processes, including pathogenic microbe infection, metabolic adaptation, cancer, acute and chronic diseases, and other stress responses. The mechanism underlying cells respond to oxygen changes to mediate subsequent signal response is the central question during hypoxia. Hypoxia-inducible factors (HIFs) sense hypoxia to regulate the expressions of a series of downstream genes expression, which participate in multiple processes including cell metabolism, cell growth/death, cell proliferation, glycolysis, immune response, microbe infection, tumorigenesis, and metastasis. Importantly, hypoxia signaling also interacts with other cellular pathways, such as phosphoinositide 3-kinase (PI3K)-mammalian target of rapamycin (mTOR) signaling, nuclear factor kappa-B (NF-κB) pathway, extracellular signal-regulated kinases (ERK) signaling, and endoplasmic reticulum (ER) stress. This paper systematically reviews the mechanisms of hypoxia signaling activation, the control of HIF signaling, and the function of HIF signaling in human health and diseases. In addition, the therapeutic targets involved in HIF signaling to balance health and diseases are summarized and highlighted, which would provide novel strategies for the design and development of therapeutic drugs.

## Introduction

Molecular oxygen is an indispensable component in mammalian cells. In the condition of normal oxygen, mammalian cell consumes oxygen and nutrients to synthesize adenosine 5’-triphosphate (ATP)^1^ It is also involved in various key biochemical reactions in the cells. Therefore, mammalian cells maintain oxygen balance to ensure their physiological function. Decreased oxygen concentration stimulates a variety of downstream signal responses in the cells. In the presence of hypoxic pressure, mammalian cells will activate a series of downstream pathways, mainly including hypoxia-inducible factor (HIF), autophagy, energy metabolic pathways like the mTOR complex 1 (mTORC1), and cell stress pathways such as ER stress;^2,3^ these pathways facilitate the cell’s response to the hypoxia stress.

The central pathway of cell response to a low oxygen environment involves HIF transcription factors, which are responsible for sensing the hypoxic environment in the cells, inducing metabolic changes, regulating cell proliferation, and controlling inflammatory response and other functions.^1,4^ Simultaneously, HIF signal is also proved the association with several diseases, such as cardiovascular, metabolic, inflammatory, and infection-related diseases.^5–7^. The discovery of this pathway provides a complete molecular framework to explicate how cells perceive oxygen changes, mediate downstream signal transduction, and provide new therapeutic targets in various human diseases.

Here, we focused on how cells recognize oxygen changes and mediate signal transduction, especially the role of HIFs in cells’ perception of hypoxia. Additionally, we comprehensively summarized the role of HIF signaling in homeostasis of cells, including the mechanism underlying upstream or downstream activation or signal transduction of HIFs, the cross-talking of HIF pathway, and other cellular pathways. Moreover, the roles of HIFs pathway in human health and diseases, and the advances and development of various drugs targeting HIFs pathway were summarized.

## History of HIF pathway

**Fig. 1:**
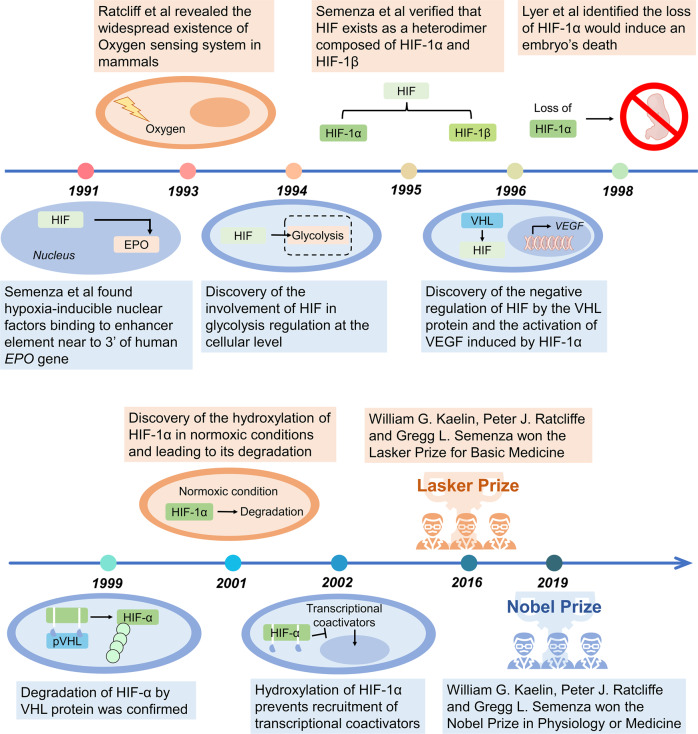
History and events of the studies on hypoxia signaling. A glance of the discoverty and advance of the knownlegment of hypoxia signaling started from 1991. In 2019, the Nobel Prize in Physiology and Medicine was awarded for the discovery of cellular mechanisms for oxygen sensing in animals

The study on HIF pathway has gained significant achievements in the past 30 years (Fig. 1). In 1991, Semenza et al. demonstrated that in the kidney or liver, hypoxic or ischemic conditions induce the production of nuclear factors that promote erythropoietin (EPO) expression by binding to the enhancer elements located 3’ to the human *EPO* gene,^8^ first reported as HIF. Ratcliffe et al. then revealed the ubiquity of this oxygen-sensing system in mammals.^9^ In their subsequent study, a regulatory effect of HIF on glycolysis was identified. Their studies uncovered that the expression of two genes associated with glycolysis, phosphoglycerate kinase (PGK) along with lactate dehydrogenase (LDHA) are elevated under hypoxia.^10^ In 1995, Semanza et al. isolated and purified HIF-1 and confirmed that HIF-1 contains two subunits: HIF-1α and HIF-1β.^11,12^ Other studies reported that HIF-1α accumulation enhances the expression of vascular endothelial growth factor (VEGF), whereas HIF-1α deficiency impairs the process of angiogenesis and eventually causes embryonic death.^13,14^

Based on the discovery of HIF function in biological process, the exact regulatory mechanism of HIF was elucidated. Kaelin et al. identified a complex formed by von Hippel-Lindau (VHL) tumor suppressor protein (pVHL) with Cullin2 (CUL2), Elongin B, and Elongin C.^15^ Among these factors, VHL protein has a negative regulatory effect on HIF,^16^ and the absence of VHL prohibits HIF degradation and promotes tumor initiation.^17^ Accumulating evidence has clarified the regulatory role of HIF. Under normoxia, HIF-1α undergoes hydroxylation to inhibit the recruitment of transcriptional coactivators,^18^ while VHL recognizes and binds to the hydroxylation sites and subsequently degrades HIF-1α.^19,20^ In the next decade 1991–2001, emerging enzymes related to HIF-1α hydroxylation are reported.^21–23^ For their contributions to the discovery of how human and animal cells perceive and adapt to oxygen supply, William Kaelin, Peter Ratcliffe, and Gregg Semenza were awarded the 2019 Nobel Prize in Physiology and Medicine.^24^

## HIFS-mediated signal transduction

### HIF family

**Fig. 2:**
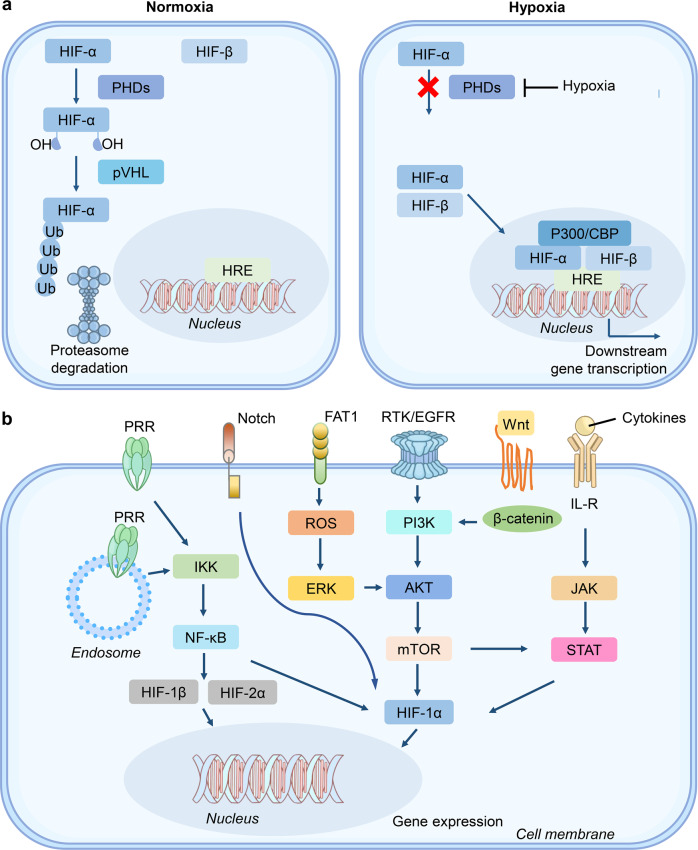
The underlying principles of hypoxia and cross-talk of HIF signal with multiple pathways. **a** Under normoxia, HIFs (*α* and *β* subunits) undergo ubiquitination mediated by PHDs (oxygen-dependent proline hydroxylase family) and pVHL (von Hippel–Lindau tumor suppressor protein). The enzymatic activity PHD is prohibited under hypoxia. HIFs are stabilized to promote downstream genes transcription. **b** The interaction among HIF signal with multiple signaling pathways

HIFs are the central factors that mediate downstream gene expression in response to hypoxic stress. The HIF family contains two different subunits: α and β. The α part composes of HIF-1α, HIF-2α, and HIF-3α; the β part contains one protein (HIF-1β). HIF-1α is widely expressed in all body tissues, while HIF-2α and HIF-3α are only detected in a few specific tissues.^25–27^ The α-subunit protein is regulated by cellular oxygen levels, whereas the β subunit is constitutively expressed.^26,28^ Under normoxic conditions, HIF-α proteins (HIF-1α, HIF-2α, and HIF-3α) undergo rapid ubiquitination and sequent degradation by proteasome through hydroxylation of prolyl residues (Fig. 2a). HIF- α proteins contain an oxygen-dependent degradation domain with two proline sites hydroxylated, by the oxygen-dependent proline hydroxylase family (PHDs), including PHD1, PHD2, and PHD3.^20,29^ Interestingly, this enzymatic activity requires oxygen, iron, and 2-oxo-glutarate.^19,29^ After hydroxylation, HIF-α interacts with pVHL and then promotes HIF-α ubiquitin-proteasome degradation.^19,30^ However, under hypoxic conditions, the enzymatic activity of PHD is inhibited, which prevents HIF-α hydroxylation and ubiquitin-mediated proteasome degradation (Fig. 2a). Subsequently, the HIF-α subunit interacts with HIF-1β to form a transcriptional complex dimerization, then entering the nucleus and combining with hypoxia-responsive elements (HREs), inducing the expression of numerous downstream genes.^31,32^ Notably, HIF-3α exerts an opposite role in the induction of hypoxia-related gene expression. Also, the abundant expression of HIF-3α reduces angiogenesis and restrains cell proliferation.^33^

### Cross-talk of pathways and HIF signal

In addition to the regulation at the protein level, multiple signaling pathways are included in the transcription of HIFs, further affecting the regulatory pathway (Fig. 2b). PI3K-mTOR signaling promotes *HIF-α* mRNA expression, suggesting its activity upstream of HIF-α.^34,35^ In addition, the upregulated PI3K-mTOR signaling in cancer cells can facilitate HIF-α activity and induce the angiogenic factors expression.^36^ Furthermore, signal transducer and activator of transcription 3 (STAT3) was phosphorylated by mTORC1 in a hypoxic environment, thereby inducing HIF-1α RNA expression.^37^ A study on T cell function showed that the activation of mTOR signal promotes HIF-α to drive metabolic reprogramming and prolongs the T cell survival.^38^ These studies indicated that PI3K-mTOR signaling regulates the mRNA level of HIF-α.

Mitochondria is a major energy metabolism organelle in a mammalian cell and the powerhouse of oxygen consumption. It plays a crucial role in the modulation of HIF-α via the enrichment of reactive oxygen species (ROS) that enhances HIF stability through inhibition of PHD function.^39,40^ Reportedly, interleukin-6 (IL-6) accelerates HIF-α expression by activating the downstream Janus kinase (JAK)-STAT3 signaling pathway,^41^ which is similar to the fact that STAT3 is phosphorylated by mTORC1, upregulating the *HIF-1α* RNA expression.^37^ In addition, the activation of pattern recognition receptors (PRRs) can trigger HIF-α transcription. The activation of the Toll-like receptor (TLR) signal drives the downstream NF-κB pathway to promote HIF-α transcription. For example, lipopolysaccharide (LPS) primes TLR4 signaling to induce *HIF-1α* mRNA expression.^42^

The ERK pathway is another important pathway that induces HIF-1α expression.^43^ Reportedly, hyperthermia promotes HIF-1α expression through AKT and ERK pathways.^44^ Besides, photodynamic therapy (PDT) induces HIF-1α expression through ROS-ERK axis, which enhances the therapy resistance.^45^ Lastly, the mitogen-activated protein kinase (MAPK) signaling activates of HIF-1α pathway through regulating the p300/CBP protein complex.^46^ These studies indicated that ERK signaling regulates the mRNA level of HIF-1α to coordinate HIF signal.

In addition to the above signaling pathways, other pathways including Wnt/β-catenin, Notch, and FAT1-ROS are also involved in HIF signals. The Wnt/β-catenin could initiate PI3K/Akt signaling and then adjust HIF-1α function.^47^ Wnt/β-catenin cooperates with HIF-1α signal in cancer cells,^48^ while HIF-1α signal also regulates Wnt/β-catenin pathway by calreticulin.^49^ Emerging studies manifest that the Notch/HIF-1α signaling modulates liver regeneration, angiogenesis, and cancer epithelial-mesenchymal-transition (EMT).^50–52^ The FAT1/ROS/HIF-1α signaling cascade is found to participate in the growth of glioblastoma (GBM).^53^

Based on the fact that mouse articular chondrocytes promoted HIF-2α expression after treatment with IL-1β, a stimulator of NF-κB pathway, NF-κB pathway could act as an activator to regulate *HIF-2α* mRNA expression in osteoarthritic.^54^ Another study found that Icariin modulated NF-κB/HIF-2α axis and reduced inflammation in chondrocyte.^55^ Since NF-κB and mTOR signaling pathways regulate the expression of HIF-1α, the above investigations imply that HIF-1α and HIF-2α may be modulated by common pathways. Although the constitutive expression of HIF-1β is independent of the cellular oxygen level,^28^ one interesting study found that NF-κB signaling also promotes HIF-1β expression.^56^

ER stress is one of the key stress pathways in the host cell in the form of cellular unfolded protein response (UPR) through activating a series of downstream factors, such as protein kinase R-like ER kinase (PERK) and activating transcription factor 6 (ATF6).^57,58^ ER stress is strongly associated with hypoxia-related pathways. HIF-1α induces ER stress response and promotes alveolar epithelial cell apoptosis.^59^ Another study revealed that HIF signaling downstream factor VEGF regulates the expression of ATF6 and PERK,^60^ suggesting a regulatory action of HIF signaling on ER stress. Besides, X-box binding protein 1 (XBP1), a key protein in UPR, is induced in a hypoxia environment and promotes tumor growth,^61^ implying that hypoxia coupled with ER stress plays certain roles in tumor development. Hypoxic pathway is recently found to interact with ER stress to affect chemoresistance in tumor development.^62^ In addition, ER stress could reduce the expression of hypoxia-related factors, such as HIFs.^63^ Therefore, the interaction between hypoxia pathway and ER stress serves an integral function in diverse biological processes.

## Biological functions of HIF

**Fig. 3:**
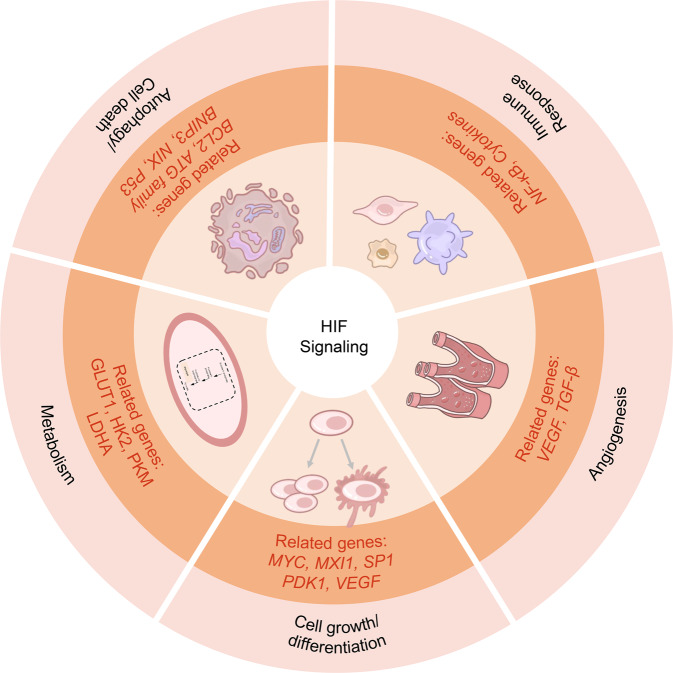
Biological functions of hypoxia signaling. Hypoxia signaling companied with the related genes participates in multiple biological processes

HIFs participate in multiple biological processes: metabolism, proliferation, cell growth and survival, glycolysis, immune response, microbe infection, tumorigenesis, and metastasis (Fig. 3). The activation of HIF-1 transcription complex induces significant gene expression,^64^ including glucose transporter 1,3 (*GLUT1,3*), *LDH-A*, *VEGF*, transforming growth factor-β (*TGF-β*), matrix metalloproteinases (*MMP*s), and nitric oxide synthase (*NOS*), which in turn play a critical part in cell metabolism, tumorigenesis, and many other aspects.^65–68^ In addition, HIF signals interact with other cellular pathways and regulate various biological processes.

### Cell metabolism by hypoxia

The generation of ATP occurs in the majority of the cells through oxidative phosphorylation. Conversely, HIF-1α stimulates PGK and LDHA in the regulation of the glycolysis process under hypoxia conditions.^10^ Anaerobic metabolism is also regulated by HIF-1α as it induces anaerobic metabolism shift through multiple enzymes related to glycolysis and glucose transporters, like pyruvate kinase M (PKM), in turn producing energy.^69^ In addition to glucose consumption and glycolysis, HIF-1α activation underlies lipid metabolism or lipid anabolism,^70–72^ effectuating its pivotal role in the liver and cardiac metabolism.

### Cell proliferation by hypoxia

Cell viability and growth are reduced due to deprivation of nutrients and dispossession of oxygen, termed hypoxia. In various cell types, such as hematopoietic stem cells, keratinocytes, lymphocytes, embryonic fibroblasts, embryonic stem cells, and a wide variety of cancer cells, hypoxia inhibits cell proliferation.^73^ HIF-1α acts biological functions in tumor proliferation and development in hypoxic conditions due to the extreme demands of energy. The tumor survival is mediated by HIF-1α in a hypoxic environment through inhibition of MYC, a transcriptional factor regulating mitochondrial mass and oxygen consumption in several human cancers. HIF-1α decreases the level of MYC by inducing the transcription of MAX interactor 1 (MXI1) (a repressor of MYC) in cancer cells and enhances mitochondrial respiration but increases the glycolysis, leading to tumor growth and survival in a low oxygen environment.^74–76^

Distinguishing to HIF-1α, HIF-2α is unable to compete with MYC for specificity protein 1 (SP1) binding through protein kinase D1 (PKD1)-mediated phosphorylation of HIF-2α.^77^ In human microvascular endothelial cells, HIF-2 α enhances SP1 activity and also facilitates MYC function to drive IL-8 expression.^78^ In primary mouse embryo fibroblasts and *VHL*^−/−^ kidney tumor cells, MYC activity is enhanced by HIF-2α.^79,80^ Moreover, HIF-2α triggers the activation of MYC by way of the stabilization of the MYC/MAX heterodimer complex under hypoxia. This effect is more exquisite than the degradation of MYC mediated by HIF-1α in cancer cells.^81^ In cancer cells, MYC regulates the HIF-2α by binding to the *HIF-2α* gene promoter and such regulation is facilitated by stem cell factors in stem cell renewal and tumor.^82^

### Hypoxia-mediated angiogenesis

HIF-1α plays a vital role in cell metabolism and physiological homeostasis.^83^ Another major function of HIF-1α is to promote angiogenesis through endothelial cell migration to a hypoxic environment by the transcription of VEGF. A new blood vessel in endothelial cells supplies oxygenated blood to a specific area.^84,85^

### Hypoxia-induced autophagy

The orchestration of multiple stress response pathways including unfolded protein response (UPR), HIF-1 signal, and autophagy, are required for the tumor cells’ adaptation and survival. Hypoxia-induced autophagy performs a certain function in tumor progression.^86^ Several hypoxia-responsive genes’ transcription is regulated by HIF-1 activation under hypoxia stress. Despite the complexities of regulation, the significance of autophagy-associated HIF-1 in tumor growth has been identified previously.^87^ Recent evidence suggested that altered expression of many HIF-1 downstream genes regulates both selective and bulk autophagy. Significantly, HIF-1 targets have been shown to have essential autophagic machinery components, such as autophagy related 5 (ATG5), ATG7, and ATG9A.^88–90^

HIF-1 could reprogram glucose metabolism by regulating a cluster of associated genes to indirectly modulate autophagy by modifying glucose metabolism.^87,91,92^ Autophagy regulates glucose uptake by controlling GLUT1 expression and function during oxygen deprivation. Upon glutamate and oxygen deprivation, PGK1 initiates autophagy via direct binding to ATGL14/VPS34/Beclin1. During tumorigenesis, glycolysis and autophagy are regulated by protein kinase activity of PGK1, which results in Beclin phosphorylation at Ser30.^93–95^ Autophagy is blocked in human T cells deficient in 6-phosphofructo-2-kinase/fructose-2,6-bisphosphatase (PFKFB3) by converting glycolysis to pentose phosphate pathway (PPP), increasing nicotinamide adenine dinucleotide phosphate (NADPH) generation and reducing ROS. On the other hand, the inhibition of PFKFB3 restricts glucose uptake in colon adenocarcinoma cells and induces autophagy.^96–98^ In acute myeloid leukemia (AML), the interaction of pyruvate dehydrogenase kinase 1 (PDK1) between unc-51-like autophagy-activating kinase 1 (UKL1) determines a regulatory manner in autophagy. The inhibition of PDK1 with dichloroacetopenone prevents this interaction and successively suppresses autophagy.^99^ Besides, hypoxia promotes the location of AKT in mitochondria, increasing phosphorylation of PDK1 on Thr346 and then inhibiting autophagy.^100^ Autophagy stimulation through hexokinases 2 (HK2)-mediated repression of TORC1 has been reported in glycose starvation neonatal rat ventricular myocytes (NRVMs).^101^ Lastly, the mTOR together with PP2A controls PHD function and further regulates HIF-1 signal and autophagy.^102^

### Hypoxia in cell death

Programmed cell death (PCD) is a common biological process in organisms that functions in the normal development of cells, maintaining tissue homeostasis against foreign infection, activating immunity, and clearing damaged cells.^103,104^ Presently, the common ways of programmed cell death include apoptosis, pyroptosis, necrosis, ferroptosis, autophagic death, and necroptosis.^105^ In addition to affecting cell proliferation, metabolic reprogramming, and autophagy, hypoxia-related pathways regulate the mode of cell death. The function of hypoxia in PCD is discussed below.

#### Apoptosis

Apoptosis is a classic way of cell death, which play a major role in plentiful biological processes that can be activated by endogenous or exogenous signals.^106,107^ To date, the role of hypoxia in apoptosis exerts a two-side effect. Hypoxia promotes cell proliferation and inhibits the occurrence of apoptosis. A study reveals that dictamnine decreases the protein expression of HIF-1α and slug to promote cell apoptosis.^108^ Besides, the HIF-1α-BNIP3 (B-cell lymphoma 2 (BCL2) and adenovirus E1B 19 kDa-interacting protein 3) pathway mediates mitochondrial autophagy to inhibit apoptosis and ROS production, exerting a protective effect in acute renal injury.^109^ In addition to HIF-1α-reduced apoptosis in hepatoma cell HepG2,^110^ HIF-2α inhibits apoptosis and autophagy of cervical cancer cells under hypoxia.^111^ Accumulating evidence demonstrated that hypoxia increases apoptosis. Typically, hypoxia reduces the proliferation of embryonic stem cells and accelerates apoptosis in response to HIF-1α knockdown.^112^ In addition, the inhibited mitochondrial function under hypoxia promotes ROS production and mitochondrial damage that accelerates apoptosis.^32^ Notably, these studies suggested that hypoxia can accelerate apoptosis independent of HIFs. Conversely, hypoxia accelerates apoptosis through HIF-dependent pathway. Several studies have identified that Nix and BNIP3, two pro-apoptotic factors, play vital roles in HIF-1 mediated apoptosis.^5,113,114^ P53 is a crucial tumor suppressor with a key role in apoptosis. HIF-1α promotes p53-dependent apoptosis.^115^ In this process, HIF-1α stabilizes p53 in dephosphorylated state and regulates p53-dependent apoptosis.^116,117^

#### Pyroptosis

A gasdermin (GSDM) family could program another type of cell death called pyroptosis,^118^ containing five members named GSDMA/B/C/D/E.^119^ Cell pyroptosis occurs after gasdermin family is cleaved by caspase or other protein, and the N-terminal pore-forming domain is located on cell membrane.^120–122^ Reportedly, hypoxia plays a key role in pyroptosis. Hou et al. demonstrated that hypoxia mediates programmed death ligand 1 (PD-L1) into the nucleus and then induces the expression of *GSDMC* gene to promote pyroptosis in tumor cells.^123^ Since the tumor microenvironment is hypoxic, pyroptosis may have varied roles in different tumors. Another study claimed that LPS induces ROS generation to promote inflammasome activation and pyroptosis in H9C2 cells.^124^ It was also confirmed that hypoxia induces ROS generation to promote pyroptosis in an NF-κB/HIF-1α-dependent pathway.^125^ Hypoxia/reoxygenation induces cardiomyocyte pyroptosis and IL-18 release, which is mediated by caspase 11-mediated cleavage of GSDMD.^126^ Strikingly, HIF-1 plays a key role in pyroptosis based on NLRP3 inflammasome.^127–130^ Based on the above findings on the role of hypoxia in inducing pyroptosis, hypoxia-induced cell death is speculated as a vital target for disease intervention.

#### Necroptosis

Necroptosis is another programmed cell death that could be regulated by hypoxia, which is mediated by cell death receptors and related to many inflammatory diseases.^131^ HIF-1α accelerated necroptosis in macrophages through miR-210 and miR-383.^132^ HIF-1α also participates in receptor interacting protein 1 (RIP1)-, RIP3-, and mixed lineage kinase domain-like protein (MLKL)-induced necroptosis and deteriorates ischemic brain injury.^133^ Conversely, a deficiency of HIF-1α and HIF-2α in the myeloid leads to macrophage necroptosis in a myocardial infarction model.^134^ These studies suggested varying roles of hypoxia-related factors in necrosis.

#### Ferroptosis

The typical character of ferroptosis is iron-dependent lipid peroxidation accumulation. Ferroptosis is associated with various diseases, including those of the intestine, kidney, liver, and tumors.^135^ Increasing evidence demonstrates a highly concerned relationship between hypoxia and ferroptosis. Fan et al. demonstrated that hypoxia restrains ferroptosis in hepatocellular carcinoma (HCC) *via* HIF-1α/solute carrier family 7 member 11 (SLC7A11) axis.^136^. Another study showed that sorafenib reduces CCl_4_-induced liver fibrosis through the induction of ferroptosis in hepatic stellate cells via HIF-1α/SLC7A11 pathway.^137^ Moreover, hypoxia stimulates SUMO/sentrin-specific peptidase 1 (SENP1) protein to promote deSUMOylation of HIF-1α in H9C2 cells, thereby inhibiting cardiomyocyte ferroptosis.^138^ Similar to the treatment of di-(2-ethylhexyl) phthalate (DEHP), exposure to MEHP (a major biometabolite of DEHP) results in HIF-1α accumulation and transfer to the nucleus, followed by activation of HIF-1α/HO-1 signaling pathway to promote ferroptosis.^139^ Altogether, hypoxia-induced cell death is speculated as a major target for disease intervention.

### Hypoxia and immune response

The immune system is an extremely complex defense system of the body, responsible for preventing pathogen invasion, recognizing and removing damaged cells, malignant cells, or other harmful components to maintain homeostasis. The immune system is mainly divided into innate and adaptive immunity. Failure to activate or excessive activation of the immune system leads to dysfunction or autoimmune diseases.^140^ In addition, the hypoxic environment is related to immune response, including innate and adaptive immunity.^141,142^ In this chapter, the role of hypoxia in immune response is summarized systematically.

#### Hypoxia in innate immunity

Innate immunity eliminates the infection, responds rapidly, and activates adaptive immunity.^142^ It is well explored that hypoxia-related factors regulate the innate immunity pathway. NF-κB is a key inflammatory response pathway that promotes HIF-α transcription.^42^ In turn, HIF-1α promotes LPS-induced NF-κB pathway activation and downstream gene expression in a succinate-dependent manner.^143^ In addition, pyruvate kinase M2 (PKM2) regulates HIF-1α function to mediate LPS-induced IL-1β expression.^144^ HIF-1α also regulates the interferon pathway. In hypoxic monocytes, HIF-1α negatively regulates the interferon expression.^145^ Upon severe acute respiratory syndrome coronavirus 2 (SARS-CoV-2) infection, HIF-1α signaling pathway activates the interferon and pro-inflammatory cytokines.^146^ In a previous study, we revealed that SARS-CoV-2 infection induces HIF-1α expression, thereby promoting viral replication and virus-induced inflammatory responses.^147^ HIF-1α is widely expressed in different innate immune cells, including macrophages, dendritic cells (DCs), and neutrophils. It also mediates metabolic reprogramming to mainly control innate immune cell activation and immune response.^148–150^

#### Hypoxia in adaptive immunity

In adaptive immune regulation, HIF-1α affects the differentiation and function of T cell-like innate immune cells, and T cells undergo metabolic reprogramming after activation. Shi et al. illustrated a vital role of HIF-1α-dependent glycolysis pathway in the differentiation of Th17 and Treg cells, whereas loss of HIF-1α reduces Th17 differentiation but enhances Treg cell differentiation.^151^ Another study showed that HIF-1 promotes the development of Th17 and inhibits the development of Tregs,^152^ implying varying glycolysis-dependence of the two cell subsets. In addition, Palazon et al. found that HIF-1α is essential for CD8^+^ T cells in anti-cancer immunity.^153^ The above studies explored that HIF exerts a regulatory role in different T cell subsets. B cell is an important adaptive immune cell. This phenomenon clarified that hypoxia plays a specific role in B cell differentiation and function in a HIF-1α-dependent glycolysis pathway.^154,155^ Additionally, HIF-1α stimulates the production of IL-10 in B cells via HIF-1α-mediated glycolysis,^156^ thus regulating B cell-related autoimmune diseases.

## Hypoxia signaling in human diseases

### Metabolic diseases

#### Hypoxia signaling in diabetes

Diabetes, a heterogeneous metabolic disease, is featured by the presence of hyperglycemia because of either defective insulin function, impaired insulin secretion or both.^157^ Diabetes is rapidly spreading worldwide, and its complications cause kidney failure, blindness, cardiovascular disease risk, and increased mortality in individuals with diabetes.^158–160^ A broad consensus was observed on four categories of diabetes: type 1 diabetes (T1D), T2D, hyperglycemia in pregnancy, and diabetes with a specific etiology that may be genetic defects or secondary to drugs, pancreatic factors, or other illnesses.^161,162^ Type 1 and T2D are primary forms of diabetes.^163^ Increasing evidence demonstrates that it is hypoxic in diabetes, wounds, pancreatic islets, and tissues (such as the kidney), indicating that hypoxia is closely involved in the occurrence of diabetes.^164–166^ Next, we described the major mechanisms underlying hypoxia signaling-regulated diabetes and diabetic complications.

Hyperglycemia is a common indicator for diagnosing T1D and T2D. High glucose levels suppress hypoxia-induced stabilization of HIF-1α protein level against degradation in specific cells.^167^ A series of studies have presented the suppressed stabilization and function of HIF-1α in the kidney, wound, and the heart of animal models of diabetes or diabetes patients.^166,168,169^ Different cell types decide specific roles of HIF-1α activity and signaling in diabetic kidney diseases. High glucose level activates HIF-1α signaling in glomerular mesangial cells,^170^ however, in proximal tubular HK-2 cells, HIF-1α signaling is suppressed by high glucose levels.^171^

Typically, activating HIF-1α signaling prevents the development of diabetic kidney disease in the T2D animal model.^172^ Inhibited HIF-1α signaling impairs wound healing, while activated HIF-1α signaling increases fibroblast proliferation, migration, and angiogenesis to promote wound healing in the diabetes animal models.^168,173,174^ Properly activated HIF-1α signaling is critical for diabetic heart disease.^175^ Pharmacologically, activating HIF-1α signaling restores the hypoxic response and improves functional recovery post-ischemia in diabetic heart diseases.^176^

Unlike HIF-1α, there are only a few studies focused on HIF-2α in diabetes. Brunt et al. suggested that overexpression of HIF-2α does not alter glucose homeostasis in pancreatic β cells.^177^ However, recent studies have described a critical role of HIF-2α in hepatic glucose homeostasis.^178,179^ Taniguchi et al. uncovered that the increased hepatic HIF-2α, but not HIF-1α, improves glucose tolerance and insulin sensitivity to ameliorate diabetes.^178^ Similarly, Wei et al. demonstrated that increasing hepatic HIF-2α ameliorates dyslipidemia, decreases hepatic gluconeogenesis, and improves glucose tolerance and hepatic insulin sensitivity in a HIF-2α-IRS-2-dependent manner.^179^

#### Hypoxia signaling in hypoglycemia

Hypoglycemia is defined by a low plasma glucose level, the development of autonomic or neuroglycopenic symptoms, and symptoms in response to the administration of carbohydrates.^180^ Interestingly, the deprivation of glucose is capable to lead to numerous cellular effects, including cell cycle arrest, autophagy, and apoptosis.^181,182^ High level of glucose can weaken HIF-1α signaling in several mammalian cell types.^183–185^ Furthermore, it is important to understand the correlation between hypoxia signaling and glucose deprivation.

Limberg et al. demonstrated that hypoglycemia-impaired cardiovascular and autonomic functions are worsened in adults with type 1 diabetes when hypoglycemia is combined with hypoxia signaling.^186^ Miro and Tirosh showed that hypoxic treatment has a strong hypoglycemic effect, and cholesterol could regulate a metabolic ketogenic shift to prevent hypoxia-induced hypoglycemia.^187^ Zamudio et al. demonstrated that altitude-induced hypoxia decreases fetal circulating glucose concentration and consumption, which unrecovered the correlation of hypoglycemia with the derivation of hypoxia-induced decline in human fetal growth.^188^

#### Hypoxia signaling in non-alcoholic fatty liver disease (NAFLD)

NAFLD is a kind of the most prevalent chronic liver disease globally,^189^ characterized by macrovesicular steatosis in hepatocytes (≥5%) in the absence of a secondary cause, such as drugs or alcohol.^190^ In the absence of overdose alcohol intake, it is a progressive disease that involves lipid accumulation and non-alcoholic steatohepatitis that ultimately causes cirrhosis and hepatocellular carcinoma.^191–193^ It is reported that the pathogenesis of NAFLD has been linked to hypoxia signaling.^194,195^ HIFs can also regulate cellular metabolism in hypoxia. HIF-1α upregulates the expression of genes encoding glycolytic enzymes (i.e., LDHA) and promotes glucose consumption, while HIF-2α represses the expression of genes associated with oxidative metabolisms (i.e., FAO) and regulates lipid storage.^70,196–199^

HIF-1α activation promotes glucose consumption and glycolysis and affects lipid metabolism.^70,71^ HIF-1α is upregulated in hepatocytes in NAFLD and is also a critical regulator of liver fibrosis in NAFLD.^200–202^ Csak et al. observed that microRNA (miRNA)-122 regulates HIF-1α in hepatocytes and is correlated with fibrosis in methionine-choline-deficient (MCD) diet-induced steatohepatitis. Wang et al. showed that palmitic acid induces HIF-1α and impairs autophagic flux and autophagy via HIF-1α in macrophages.^203^ HIF-1α also mediates activation of NF-κB and production of monocyte chemoattractant protein-1 (MCP-1), impairs autophagy, and increases IL-1β production. Both MCP-1 and IL-1β contribute to MCD diet-induced non-alcoholic steatohepatitis.^203^ Asai et al. showed that cholesterol induces HIF-1α activation and liver steatosis, and HIF-1α reduces the expression of hepatic aquaporin 8 (AQP8) and promotes cholesterol gallstone formation.^204^ The high expression of hepatic HIF-1α is observed in the livers of patients with NAFLD and gallstones than in those without gallstones.^204^

HIF-1α and −2α affect lipid metabolism; however, HIF-2α is the predominant subunit regulating lipid metabolism, which suppresses fatty acid oxidation and promotes the genes related to fatty acid synthesis and lipid storage.^194,205^ Knockdown of HIF-2α protein reverses lipid metabolism dysregulation by acute hypoxia in the human hepatocellular carcinoma HepG2 cell line.^206^ Rankin et al. demonstrated that constitutive HIF-2α activation impairs fatty acid β-oxidation and increases lipid storage capacity, leading to severe fatty liver disease in mice.^205^ Morello et al. found that HIF-2α activation influences the severity of steatohepatitis and fibrogenesis in human NAFLD by upregulating the expression of histidine-rich glycoprotein (HRGP).^194^ Qu et al. revealed that HIF-2α activation promotes the developmental progression of steatohepatitis by increasing lipid accumulation, subsequent inflammation, and eventually fibrosis.^207^

#### Hypoxia signaling in osteoporosis

Osteoporosis, a common skeletal disease is featured by systemic impairment of bone mass, strength, and microarchitecture, which increases the risk for fragility fractures.^208^ Oxygen is required for the activity of skeletogenic cells and many fundamental cellular processes that are critical for normal fracture healing.^209^ In recent years, several studies elucidated the mechanisms by which HIFs (HIF-1α and HIF-2α) impact bone remodeling and pathologies.^210^ However, the underlying correlations between hypoxia signaling and osteoporosis remain poorly understood.

Miyauchi et al. showed that estrogen receptor α (Erα) decreases HIF-1α protein levels in osteoclasts, and osteoclast formation is blocked by HIF-1α deficiency in hypoxic conditions.^211^ Importantly, HIF-1α is controlled by estrogen signaling in osteoclasts, and thus, it may be a promising therapeutic target to treat postmenopausal osteoporosis.^211^ Tando et al. illustrated that mouse HIF-1α protein accumulates in osteoclasts following orchidectomy in vivo and in osteoclasts cultured in hypoxic conditions in vitro.^212^ The protein level is suppressed by testosterone treatment in osteoclasts cultured in hypoxic conditions, and HIF-1α inhibitor abrogates testosterone deficiency-induced bone loss and osteoclast activation in orchidectomized mice.^212^ This testosterone deficiency accelerates HIF-1α protein accumulation, thereby promoting the development of male osteoporosis.^212^ Zhao et al. suggested that the expression of HIF-1α and HIF-2α was suppressed by pVHL in osteoblasts, and HIF signaling activation in osteoblasts might prevent the bone loss induced by ovariectomy and increased angiogenesis and osteogenesis in mice.^213^ Hence, HIF-1α protein may be a critical therapeutic target for osteoporosis.^211–213^

### Infectious diseases

#### Hypoxia and infectious pneumonia

**Fig. 4:**
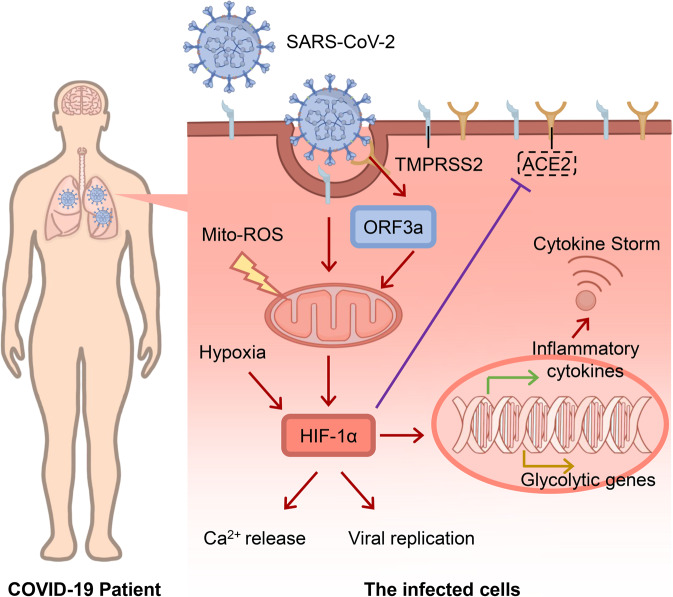
Role of HIF-1α in hypixa signaling in COVID-19. When SARS-CoV-2 entering host cells, viral ORF3a protein induces HIF-1α expression through triggering mitochondrial reactive oxygen species (ROS) activation. The accumulated HIF-1α stimulates Ca^2+^ release, promotes viral replication and enhances glycolytic and inflammatory genes, which leads to a cytokine storm

Infectious pneumonia is an acute inflammation of the lung tissue caused by large-scale pathogens including viral and bacterial infections.^214^ Patients confirmed with infectious pneumonia are at a high risk of acute lung injury (ALI), especially those with specific types of viral pneumonia,^215^ including *Streptococcus pneumoniae* (*S. pneumoniae*), the most common cause of pneumonia, and influenza virus, frequently leading to viral pneumonia. Notably, *S. pneumoniae* usually infects nervous system to cause fatal bacterial meningitis, and the course of the infection could be affected by hypoxia and HIF-1.^216^ Hypoxia is the hallmark of SARS-CoV-2 pneumonia.^217^ Therefore, hypoxia signaling might be closely associated with the occurrence and progression of SARS-CoV-2 pneumonia. Herein, we described the correlation between coronavirus disease 2019 (COVID-19) and hypoxia signaling (Fig. 4).

Serebrovska et al. speculated that the activation of HIF-1α decreases the expression of angiotensin converting enzyme-2 (ACE2) along with transmembrane serine protease 2 (TMPRSS2) while increasing the expression of ADAM metallopeptidase domain 17 (ADAM17) on the surface of alveolocytes under hypoxic conditions, thereby decreasing the invasiveness of SARS-CoV-2.^218^ The study also concluded that HIF-1α signaling participates in severe hypoxia-induced activation of pro-inflammatory cytokine expression and cytokine storm phase of COVID-19.^218^ We have recently revealed that SARS-CoV-2 induces expression of HIF-1α and secretion of inflammatory cytokines *via* ORF3a, and conversely, HIF-1α facilitates SARS-CoV-2 replication and aggravates inflammatory responses.^147^ HIF-1α also facilitates the infections of other viruses, such as herpes simplex viruses 1 (HSV-1) and vesicular stomatitis virus (VSV).^147^ Codo et al. showed that SARS-CoV-2 triggers mitochondrial ROS production, which enhances HIF-1α stabilization and sustains SARS-CoV-2 replication in monocytes.^219^ Mitochondrial ROS-mediated stabilization of HIF-1α also sustains replication of SARS-CoV-2 in monocytes.^219^ However, Prieto-Fernández et al. have shown that hypoxia reduces the binding of the SARS-CoV-2 spike (S) protein to epithelial cells through decreasing ACE2, neuropilin-1 (NRP1), and cellular heparan sulfate (HS) expression.^220^

#### Hypoxia and viral hepatitis

The term viral hepatitis means liver inflammation induced by hepatic viral infections of mainly hepatitis B virus (HBV) and hepatitis C virus (HCV).^221^ Viral hepatitis is a global public health problem that leads to thousands of patients dying of acute and chronic infections, liver cirrhosis, and cancer.^222^ In 2000, Lee et al. demonstrated that the expression of HBV X protein (HBx) was elevated when HBV-infected hepatoma cells were cultured under hypoxic conditions. Concurrently, when a reporter plasmid carrying HBV Enh1 was transfected into hepatoma cells under hypoxia, the HBV enhancer 1 (Enh1) activity was augmented.^223^

In hepatocarcinogenesis, HBx protein may be a critical mediator of hypoxia-induced angiogenesis.^223^ It increases the transcriptional and translational level and also stabilizes HIF-1α.^224,225^ Moreover, HBx promotes the HIF-1α transcription by activating MAPK pathway.^226^ Yoo et al. have shown that HBx protein increases the transcriptional level of metastasis associated 1 (MTA1) and histone deacetylase 1 (HDAC1), thereby enhancing HIF-1α protein in hepatocellular carcinoma cells.^227^ HBV also induces the HIF-2α expression via HBx protein, conversely, HBx activates NF-κB signaling to increase HIF-2α expression.^228^

Hallez et al. found that DNase I, a cellular restriction factor of HBV, is induced by HIF-1α.^229^ Wing et al. found that HIF-1α and HIF-2α promote HBV replication *via* activating the HBV basal core promoter.^230^ HIF1α stabilization offers a reservoir for HBV in immune-active patients and impairs NF-κB-mediated A3B induction, which is critical for eliminating HBV covalently closed circular DNA (cccDNA).^231^ Consequently, HIF-1α is a potential target in anti-HBV strategy in the context of immune-mediated A3B induction.

Furthermore, Ripoli et al. showed that HCV protein expression stabilizes HIF-1α under normoxic conditions, and glycolytic enzymes are upregulated by activated HIF-1α in HCV-infected cells.^232^ Under hypoxic conditions, HCV core protein enhances HIF-1α protein expression, which then elevates VEGF expression.^233^ Zhu et al. found that HCV core protein enhances the HIF-1α expression and stabilization, and subsequently, HIF-1α stimulates VEGF expression in Huh7.5.1 cells.^234^ Both VEGF and HIF-1α are crucial angiogenic factors. Hence, HIF-1α might be a new therapeutical target against HCV-induced HCC.^234^

Apart from the above bacterial and viral infection, hypoxia is found to be closely related to the pathogenesis of multiple neurological infectious diseases, including enterovirus, mumps, lymphocytic choriomeningitis, and type I and II scab viruses,^235^ the interconnection between hypoxia and infectious diseases in nervous system is taken under consideration to a potential targeted therapy in the following investigations.

### Neoplastic diseases

#### Hypoxia in colon cancer

**Fig. 5:**
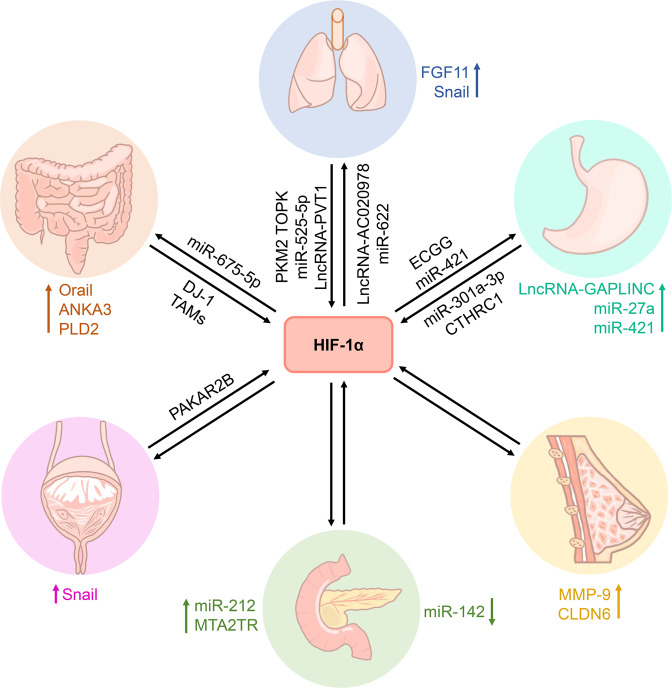
Summarized paticipation of HIF-1α in the tumorgenesis. The roles of HIF-1α in various kinds of human cancer. The tumorgenesis arises by the regulation of HIF-1α with intermediator and effectors such as indicated protein, miRNAs, or lncRNAs

Colon cancer is one of the most common cancers worldwide, with the highest mortality rate along with breast, lung, and prostate cancers.^236^ The colon and rectum are the final portions of the human digestive tract. Colon cancer arises from the colonic epithelial cells that line the lumen of the organ and results from a multistep process of colon neoplasia over several years.^237^ Hypoxia is a typical feature of solid tumors in common and it is related to the progression and metastasis of colon cancer.^238–240^ For example, the expression of Orai1 is induced by hypoxia in colon cancer, which promotes hypoxia-induced invasion and angiogenesis.^241^ The correlation between colon cancer and hypoxia is illustrated (Fig. 5).

HIF-1α was upregulated in colon cancer tissues.^242^ Santoyo-Ramos et al. showed that HIF-1α and HIF-2α are expressed in human colon cancer cells but not in non-malignant cells under normoxic conditions.^243^ Jeon et al. revealed that protein S-glutathionylation increases the protein level of HIF-1α in hypoxic colon cancer cells.^244^ Zheng et al. demonstrated that DJ-1 protein facilitates the survival of human colon cancer cells by the increased HIF-1α protein expression by means of PI3K-AKT signaling pathway.^245^

Under hypoxic stress, upregulated HIF-1α induces the expression of phospholipase D2 (PLD2) in colon cancer cells, while downregulation of the protein significantly reduces the expression of PLD2 and tumor volume.^238^ Hypoxia-induced elevated expression of PLD2 facilitates cell proliferation by NF-κB signaling activation to upregulate the expression of Cyclin D1 in colon cancer.^246^ Du et al. have suggested that annexin A3 (ANXA3) expression is upregulated by HIF-1α under hypoxic stress and promotes tumor growth in colon cancer.^247^ The expression of HIF-1α and semaphorin 4D (Sema4D) is closely related to lymphatic metastasis and specific histological types in colon cancer. Mechanistically, in colon cancer, tumor-associated macrophages (TAMs) may accelerate cell migration and invasion *via* upregulation of HIF-1α and Sema4D.^248^ Costa et al. found that miR-675-5p is overexpressed in metastatic colon cancer patients and is involved in tumor progression by promoting HIF-1α-induced EMT.^249^ HIF-1α mediates hypoxia-induced apoptosis-inducing factor (AIF) inhibition, and downregulation of AIF contributes to hypoxia-induced EMT of colon cancer.^250^ In a subset of colon cancers, HIF-1α is a positive factor for non-hypoxia-mediated cell proliferation in vitro and in vivo, and hypoxia-mediated cell proliferation and survival in vitro but does not contribute to the hypoxic tumor compartments in vivo.^251^

HIF-2α is essential in the inflammatory response and the regeneration and proliferation capacity of the intestine following an acute injury, and its chronic activation enhances the proinflammatory response, intestinal injury, and colorectal cancer.^252^ Franovic et al. showed that suppression of HIF-2α restrains tumorigenesis and the proliferation of genetically diverse human cancer cells in vivo.^253^ Xue et al. suggested that HIF-2α activation increases tumor progression in colon cancer, whereas the HIF-2α-induced tumor formation is reduced upon low-iron treatment.^254^

Experimental evidence highlighted that apart from human colon carcinoma cell lines, HIF-2α is also important for the survival of patient-derived primary colon cancer cells.^255^ Different from HIF-1α, HIF-2α plays an important role in resistance in colon malignant cells.^255^ Cyclooxygenase 2 (COX2) expression is dependent on HIF-2α in colon tumors, and its inhibition reduces HIF-2α-induced colon tumor formation.^256^ Yes-associated protein 1 (YAP1) activity is upregulated by HIF-2α in CRC-derived cell lines and mouse models; HIF-2α also promotes colon cancer growth by upregulating the activity of YAP1.^257^

#### Hypoxia signaling in lung cancer (LC)

LC, a kind of malignant tumor and a leading cause of death worldwide, is mostly classified into two categories, namely small cell lung cancer (SCLC) and non-small cell lung cancer (NSCLC).^258–260^ NSCLC is the major subtype of LC and accounts for about 80% of all patients with LC.^261^ The initiation of LC derives from a highly vascularized and oxygenated tumor microenvironment, crucial for tumor progression.^262,263^ Current studies have found that hypoxia signaling is associated with multiple processes in the occurrence and progression of NSCLC and SCLC,^264,265^ which are controlled precisely and differentially (Fig. 5).

Hypoxia elevates the HIF-1α level in LC cells.^266^ Moreover, HIF-1α expression in LC is higher than in normal lungs. NSCLC patients have a higher HIF-1α expression than SCLC patients, while upregulation of HIF-1α is closely related to tumor growth and survival rate of NSCLC.^267–269^ It is reported that long non-coding RNA (lncRNA) PVT1 increases the expression of HIF-1α in NSCLC.^270^ Wu et al. found that fibroblast growth factor 11 (FGF11) is upregulated in NSCLC tumor tissues and cell lines, and high expression of FGF11 is related to a poor prognostic outcome in NSCLC patients.^271^ miR-525-5p negatively regulates FGF11 while FGF11 promotes the expression of HIF-1α for NSCLC progression.^271^ On the other hand, T-lymphokine-activated killer cell-originated protein kinase (TOPK) positively regulates HIF-1α expression and promotes Snail expression, leading to EMT and invasion of NSCLC.^272^ In response to hypoxia, elevated lncRNA-AC020978 accelerates proliferation and the glycolytic metabolism of NSCLC by regulating PKM2-enhanced HIF-1α transactivation activity.^273^ Overexpression of miR-622 mediated by forkhead box O3 (FOXO3a) represses HIF-1α to hinder the migration and invasion of LC cells.^274^ Gamma linolenic acid (GLA) inhibits hypoxia-driven proliferation and invasion of NSCLC cells by inhibition of HIF-1α-VEGF pathway in vitro.^275^ Subsequently, HIF-1α inhibition suppresses the hypoxia-induced EMT phenotype and increases the efficacy of immune checkpoint blockade in the treatment of NSCLC.^276^

The study of the correlation between HIF-2α and LC has not been elucidated clearly. Kong et al. showed a higher expression of nuclear paraspeckle assembly transcript 1 (NEAT1) in NSCLC tissues and cells than that in normal controls, and NEAT1 knockdown suppresses cell proliferation, migration, and invasion in NSCLC.^277^ Interestingly, NEAT1 promotes EMT and NSCLC cell metastasis under hypoxia in a HIF-2α-dependent manner.^277^ Wang et al. demonstrated that lncRNA HIF2PUT was downregulated in NSCLC tissues and cell lines, and its overexpression inhibits NSCLC proliferation and invasion via HIF-2α pathway.^278^

#### Hypoxia signaling in gastric cancer (GC)

GC is a high concern for health globally and the second cause of cancer deaths after LC.^279^ The causes of GC are multifactorial, although *Helicobacter pylori* infection is considered the main cause; its effects are modulated by environmental, microbial, and host factors.^280^ Hypoxia is closely related to the aggressive tumor phenotypes of gastric carcinomas,^281,282^ including the metastatic ability of cancer cells.^283,284^ For example, hypoxia increases GC malignancy partially through transcriptional activation of lncRNA-GAPLINC in a HIF-1α-dependent manner.^285^ Therefore, the factors underlying the correlation between GC and hypoxia need to be investigated further (Fig. 5).

HIF-1α overexpression is a poor prognostic indicator for patients with GC and is highly correlated with histology, depth of invasion, and microvessel density.^286^ HIF-1α stimulates multidrug resistance in GC cells through stimulating the transcription of miR-27a.^287^ HIF-1α-induced miRNA-421 promotes metastasis, inhibits apoptosis, and induces cisplatin resistance by targeting E-cadherin and caspase-3 in GC.^288^ Liu et al. suggested that HIF-1α and Wnt/β-catenin signaling pathways promote the invasion of hypoxic GC cells.^48^

Hypoxia increases the migration and invasion of GC cell line BGC-823 by activating HIF-1α and inhibiting N-myc downregulated gene 2 (NDRG2)-associated signaling pathway.^289^ Xia et al. demonstrated that hypoxia promotes the release of GC exosome and the expression of miR-301a-3p; then, miR-301a-3p-rich exosomes increase HIF-1α accumulation and promote GC malignancy and metastasis.^290^ Ding et al. showed that collagen triple helix repeat containing 1 (CTHRC1) overexpression increases cell migration and invasion capacity in GC. CTHRC1 upregulated the expression of HIF-1α to increase CXC chemokine receptor 4 (CXCR4) expression, ultimately promoting cell migration and invasion.^291^ Epigallocatechin gallate (EGCG) induces apoptosis and impedes proliferation in GC SGC7901 cells by downregulating the expression of HIF-1α and VEGF under hypoxia.^292^ Downregulation of HIF-1α, leading to suppressing the PI3K/AKT pathway and VEGF expression, might inhibit the proliferation, migration, and invasion of GC.^293^

#### Hypoxia signaling in breast cancer (BC)

BC is the most common malignant tumor diagnosed in women.^294^ It is also the leading cause of cancer-related deaths in women globally.^279^ Hypoxia signaling serves an essential role in BC and an increased level of HIF-1α has been documented in BC.^295^ Overexpression of HIF-1α is significantly associated with poor disease-free and overall survival in BC patients.^296^ Sun et al. have shown that HIF-1α is closed to tumor differentiation, lymph node metastasis, and clinical stage with respect to survival in BC patients.^297^ Next, the correlation between BC and hypoxia was interpreted comprehensively (Fig. 5).

HIF-1α overexpression effectuates via different regulatory pathways in BC: (a) hypoxia induces perinecrotic HIF-1α overexpression with a robust expression of hypoxia-related genes that are responsible for poor prognosis; (b) normoxia induces diffuse HIF-1α overexpression lacking major hypoxia-associated downstream effects, which is a favorable prognosis.^298^ Marton et al. showed that HIF-1α overexpression indicates an unfavorable prognosis and could serve as an additional prognostic factor in neuroendocrine BCs.^299^ Dales et al. demonstrated that mRNA expression of HIF-1αTAG splice variant reflects a stage of BC progression and is related to poor prognosis.^300^ Hoffmann et al. found that hypoxia promotes BC cell invasion through HIF-1α-mediated upregulation of cysteine-rich protein 2 (CSRP2), an invadopodia actin-bundling protein.^301^ Choi et al. suggested that HIF-1α promotes the MMP-9 expression under hypoxic conditions, which affects BC cell invasion.^302^ HIF-1α signaling is critical in ATP-driven chemoresistance and may serve as a potential target for BC therapies.^303^

BC cells display phenotypic diversity in response to hypoxic or normoxic microenvironments. HIF-1α induces the expression of hematopoietic pre-B cell leukemia transcription factor-interacting protein (HPIP) that establishes cell survival and promotes migration and invasion of cells, EMT, and metastatic phenotypes under hypoxia. Accumulation of HPIP stabilizes HIF-1α to support cell growth.^304^ Jia et al. demonstrated that claudin 6 (CLDN6) functions as a tumor suppressor in BC and is upregulated by HIF-1α under hypoxia.^305^ Increased CLDN6 weakens the stability of HIF-1α protein by reducing the expression of SENP1 and preventing the deSUMOylation of HIF-1α; the negative feedback loop slows down the hypoxia-induced BC metastasis.^305^ Hypoxia-responsive miR-141-3p is involved in the progression of BC, which prevents hypoxia-induced BC by inhibiting the high mobility group box 1 (HMGB1)/HIF-1α signaling pathway.^306^ Breast cancer metastasis suppressor 1 (BRMS1), a novel metastasis suppressor protein without the activity of anti-proliferation, attenuates TGF-β1-induced EMT and invasion of BC cells through suppressing HIF-1α expression.^307^

Similar to HIF-1α, Wang et al. suggested that HIF-2α expression is significantly correlated with tumor size, lymph node involvement, and metastasis, and high expression of the protein is associated with poor overall survival in BC patients.^308^ Thus, HIF-2α could be a valuable biomarker of BC progression and patient survival.^308^ It may promote the migration and invasion of human BC MCF-7 cells under hypoxic conditions by potentiating the Notch3 pathway.^309^ Bai et al. revealed that the downregulation of HIF-2α suppresses the stemness of human BC MDA-MB-231 cells and promotes apoptosis.^310^

#### Hypoxia signaling in pancreatic cancer

Pancreatic cancer is a fatal malignancy, predominantly seen in men at an advanced age of 40–85 years. It ranks first among asymptomatic cancers.^311^ Pancreatic cancer is extremely difficult to detect as it lacks early signs and spreads rapidly to the surrounding organs.^311^ The high malignancy of pancreatic cancer is mostly attributed to the hypoxic tumor microenvironment.^312,313^ Pancreatic cancer is accompanied by HIF-1α overexpression.^314,315^ Herein, we summarized the mechanism by which hypoxia signaling affects the tumorigenesis and progression of pancreatic cancer (Fig. 5).

HIF-1α is overexpressed in pancreatic cancer patients, and it regulates expression of various genes associated with pancreatic cancer.^315,316^ HIF-1α overexpression induces EMT in an NF-κB signaling pathway-dependent manner.^317^ Several findings discovered that high expression of HIF-1α significantly enhances the capacity of anti-apoptosis in pancreatic cancer cells.^318,319^

Upregulation of autophagy induced by HIF-1α improved the malignancy of pancreatic cancer through potentiating EMT and migration of pancreatic cancer stem cells.^320^ Yue et al. showed that HIF-1α facilitates the expression of miR-212 and results in the development of pancreatic ductal adenocarcinoma.^321^ Zeng et al. demonstrated that MTA2 transcriptional regulator lncRNA (MTA2TR) is overexpressed in pancreatic cancer patient tissues compared to paired noncancerous tissues and promotes pancreatic cancer cell proliferation and invasion in vitro and in vivo.^322^ MTA2TR is transcriptionally regulated by HIF-1α under hypoxic conditions.^322^ Furthermore, miRNAs regulate HIF-1α on the EMT of pancreatic cancer cells. The level of miR-142 was obviously lower in pancreatic cancer cell lines and tissues than that in normal tissues. Downregulating the expression of miR-142 increases HIF-1α expression to upregulate EMT-related proteins, eventually enhancing the invasion and migration of pancreatic cancer cells.^323^

Wang et al. showed that the mRNA levels of *HIF-1α* and *HIF-2α* were upregulated in pancreatic cancer. However, their protein expression patterns differed markedly with varied roles in pancreatic cancer.^324^ HIF-1α serves as an unfavorable prognostic indicator, whereas HIF-2α is a favorable prognostic indicator in pancreatic cancer patients.^324^ MiR-301a was upregulated by HIF-2α-dependent signaling pathway, and it promotes hypoxia-induced EMT of pancreatic cancer cells.^325^ Yang et al. suggested that HIF-2α promotes EMT by regulating Twist2 binding to the E-cadherin promoter in pancreatic cancer.^326^ HIF-2α facilitates the formation of vasculogenic mimic in pancreatic cancer by regulating Twist1 binding to VE-cadherin promoter.^327^

#### Hypoxia signaling in prostate cancer

Prostate cancer is a major disease in males around the world.^328^ It is the second most common form of cancer in men, surpassed only by nonmelanoma skin cancer.^328^ The incidence and mortality of prostate cancer are correlated with the mean age at diagnosis is 66 years.^329^ Zhong et al. found that expression of HIF-1α increases in human and rat prostate cancer cell lines.^330^ Hypoxia signaling plays a vital role in the tumorigenesis and progression of prostate cancer. Herein, we illustrated the complex correlation between prostate cancer and hypoxia (Fig. 5).

Hypoxia significantly enhances the invasiveness of prostate cancer PC3 cells by upregulating HIF-1α expression and autocrine tumor necrosis factor (TNF)-α production.^331^ HIF-1α cooperates with TNF-α and stabilizes Snail, which in turn upregulates the invasiveness-associated genes, *MMP9*, fibronectin, and vimentin.^331^ Moreover, HIF-1α expression is associated with an increased risk and clinicopathological significance in prostate cancer patients.^332^ Xia et al. revealed that protein kinase CAMP-dependent type II regulatory subunit beta (PRKAR2B) increases HIF-1α expression, a key mediator of the Warburg effect.^333^ Interestingly, PRKAR2B-HIF-1α loop enhances the Warburg effect that provides a growth advantage in prostate cancer.^333^

### Cardiovascular diseases

Cardiovascular diseases are the leading threat to life and health worldwide.^334,335^ The circulatory system, i.e., the organs and tissues in the body that carry blood, primarily the heart and blood vessels (arteries, veins, and capillaries), is involved in the series of illnesses.^336,337^ Hypoxia is one of the most important pathogenic factors of cardiovascular diseases.^338–341^ It heralds the onset of many cardiovascular diseases, i.e., arteriosclerosis, pulmonary hypertension, and heart failure.^342^ The occurrence and development of cardiovascular diseases can be induced by sympathetic excitation disorder, oxidative stress, inflammatory response, endothelial injury, abnormal glucose, and lipid metabolism caused by hypoxia.^343–346^

HIF-1α is the primary controller of physiological and pathological hypoxia and is widely expressed in cardiovascular diseases.^141,216,347^ Almost all genes related to hypoxia, including glucose transporter (*GLUT*), *VEGF*, glycolytic enzymes, cell survival factors, and cell surface receptors, are directly or indirectly regulated by HIF-1.^348^ The levels of HIF-1α subunits increase exponentially with the decrease in oxygen concentration to regulate hypoxic adaptive response.^349^ During an oxidative stress response, ROS promotes HIF-1α expression to activate the transcription of several genes, such as endothelin-1 (ET-1); the expression of ET-1 contributes to cardiovascular diseases.^350^ Previous studies have shown that the expression of HIF-1α activates a series of profibrotic transcriptional genes, including collagen I, III, IV, and lysyl oxidase, leading to myocardial fibrosis.^351–354^ The different expressions of HIF-1α in the cardiovascular cell system, significantly affect the function of these cells and performing a certain part in the diseases including atherosclerosis, pulmonary hypertension, cardiomyopathy, arrhythmia, and congenital heart disease.

#### Hypoxia in atherosclerosis

Atherosclerosis, as the primary cause of cardiovascular disease, leads to mortality and disability worldwide. It is characterized by chronic inflammatory changes in large and medium-sized arterial walls,^355^ including lipid deposition, atheromatous plaque formation and rupture, inflammatory cell infiltration, and endothelial function damage.^356,357^ The formation mechanism of atherosclerosis includes oxidative stress, arterial endothelial injury and dysfunction, foam cell formation, and subsequent lipid deposition and thrombosis.^358^ Arteriosclerosis begins with endothelial dysfunction that induces mononuclear cell infiltration.^359^ Cytokines released by mononuclear cells stimulate the proliferation of smooth muscle cells in the media of blood vessels and the new intima.^360^ In addition, mononuclear cells activate into macrophages, during which smooth muscle cells of the new intima ingest lipids to become foam cells, forming atheromatous plaques.^361,362^

Atherogenesis is related to hypoxia. Under such conditions, the extracellular nutrients and lipids induce the formation of hypoxic areas in arterial plaques, especially in macrophages, vascular smooth muscle cells, and endothelial cells.^363,364^ These cells subsequently express HIF in response to hypoxia.^364^ HIF-1α is expressed in 49% of carotid and 60% of femoral endarterectomy patients, providing evidence of its involvement in atherogenesis.^365^ In addition, pimidazole is increased in hypoxic zones of atherosclerotic areas, indicating the involvement of hypoxia in atherogenesis.^366^ ATP-binding cassette transporter A1 (ABCA1) and apolipoprotein A1 (ApoA-1) contribute to monocyte-macrophage infiltration and lipid deposition with plaque formation in the arterial wall, respectively.^367^ HIF-1α interacts with NF-κB and promotes the expression of ABCA1 to exert an anti-atherosclerotic role in the pathogenesis of atherogenesis in THP-1.^368^ Once the oxygen concentration in the cells is low, HIF-1α signaling participates in the formation and rupture of atherosclerotic plaques by promoting the expression of VEGF.^13^ Subsequently, VEGF stimulates neovascularization, promotes atherogenesis, increases plaque instability, and hastens plaque rupture.^13^

In human vascular smooth muscle cells, the expression of low-density lipoprotein receptor-related protein (LRP1) was upregulated by HIF-1α, promoting the deposition of lipids in plaques.^369^ Furthermore, lncRNAs are differentially expressed in patients with non-ST-segment elevation myocardial infarction (NSTEMI) and ST-segment elevation myocardial infarction (STEMI) through the HIF-1α signaling pathway, which might become a serological marker to distinguish between NSTEMI and STEMI.^370^ Previous studies have shown that HIF-1α and HIF-2α are increased in atherosclerosis, and lesions aggravate with the increase in HIF.^364^ Moreover, in a high-fat diet mice model, the selective deficiency of HIF-1α in endothelial cells relieved the lesion formation in 6 weeks.^371^ In apolipoprotein E knockout mice (ApoE^−/−^) mice, reduced HIF expression decreased VEGF activity and intimal hyperplasia.^372^. Furthermore, the deletion of *Hif-1α* gene in ApoE^−/−^ mice reduced the atherosclerotic lesions, inflammation, and the level of chemokines by upregulating miRNA-19a.^371^ Folco et al. demonstrated that when exposed to hypoxia, human macrophages and foam cells had increased glucose uptake, especially in macrophage-rich regions of the plaques.^373^ The studies showed various regulations of atherosclerosis by HIF in different types of cells, although the underlying mechanism needs to be further investigated.

#### Hypoxia in pulmonary hypertension (PH)

Pulmonary hypertension (PH) is characterized by hypoxia-induced pulmonary vessel contraction, vascular remodeling, and increased pulmonary circulation resistance, which results in elevated pulmonary artery pressure.^374^ Subsequently, the disrupted pulmonary artery endothelial cells (PAECs) produce substances that induce smooth muscle cell proliferation, resulting in neointima development and increased arterial thickening in PH. Compared to healthy controls, proliferating PAECs generate more vasoconstrictors while producing less nitric oxide (NO) and prostacyclin.^375^ However, the underlying mechanism is yet unknown. Reportedly, HIF is associated with the pathophysiology of PH. Both heterozygous HIF-1-deficient and HIF-2-deficient mice are protected from chronic hypoxia-induced PH.^376,377^ The occurrence and development of PH are influenced by inducible nitric oxide synthase (iNOS) and ET-1.^378,379^ HIF-1 activates and boosts the expression of iNOS and ET-1 under hypoxia,^380,381^ which might underlie the mechanism of PH.

One of the primary enzymes involved in endothelial cell (EC) proliferation and pulmonary dilation of blood vessels is arachidonate 5-lipoxygenase (ALOX5).^382^ When human PAECs are exposed to hypoxia, ALOX5 pathway is activated, increasing H_2_O_2_ generation and contributing to H_2_O_2_-dependent EC proliferation.^382^ Furthermore, Su et al. found that *ALOX5* promoter harbors the potential binding sites for early growth response protein 1 (EGR1) and SP1; both act as coregulators of erythropoietin receptor expression in LC cells in collaboration with HIF.^383^ Moreover, glucose absorption in idiopathic PAH (IPAH) patients’ lungs and the ECs is dramatically elevated with the decrease in mitochondrial concentration in EC and the increase of EC proliferation,^384–386^ while knockdown of glycolytic regulator PFKFB3 protects the mice against hypoxia-induced PH.^384^ Consequently, HIF in ECs’ physiology might play a role in PH formation. Notably, the mutual regulation of CD146 and HIF-1α is a key factor in the pathological mechanism of vascular reconstruction, remodeling, and PH formation.^387^ In addition, CD146 and HIF-1α promote each other’s expression and accelerate vascular remodeling and PH formation.^387^ Therefore, the regulation of HIF expression might be a potential target for the treatment of PH.

#### Hypoxia in cardiomyopathy

Cardiomyopathy is a category of disorders that produces anatomical and functional problems in the heart. It is classified as primary or secondary, with diverse phenotypes, such as dilated, hypertrophic, or restricted.^388^ However, the prevalence and progression of cardiomyopathy are not well understood. Chen et al. demonstrated that HIF-1α and FoxO3a collectively contribute to increased expression of the death factor BNIP3 and promote cardiac cell apoptosis in response to a combined stimulation of high glucose plus hypoxia.^389^ Hypoxia-induced mitogenic factor (HIMF) overexpression increases HIF-1α in neonatal rat cardiomyocytes, confirming the role of HIMF in myocardial hypertrophy. Thus, the deletion of HIF-1α reduces cardiomyocyte hypertrophy produced by HIMF and suppresses myocardial hypertrophy, making it a potential target for myocardial hypertrophy therapy.^390^ Reportedly, HIF-1α and PPAR are major regulators of glycolysis and lipid anabolism; the expression of these molecules is increased in hypertrophic cardiomyopathy. Also, these molecules jointly regulate and participate in the changes in cardiac metabolism, whereas HIF-1 accumulation is limited to pathological cardiac hypertrophy, but not physiological hypertrophy, in humans and mice.^72^ Some studies demonstrated that long-term intermittent hypoxia (IH) exposure causes continual activation of HIF-1α, which is responsible for the rise in infarct size.^391,392^ However, sustained heart-specific HIF-1α overexpression is beneficial in mice in the short term, causing cardiac insufficiency with age.^393^ An increased HIF-1α expression is detected in cardiac samples from cardiomyopathy patients, but a high level of plasma HIF-1α in patients with decompensated heart failure is related to low ejection fraction and survival.^393–395^ Taken together, the current study focuses on HIF-1α in primary cardiomyopathy, which demonstrates that HIF-1α has negative consequences, but its role and mechanism in secondary cardiomyopathy require further exploration.

#### Hypoxia in arrhythmia

Arrhythmia is an irregular frequency and/or rhythm of heartbeat ascribed to the origin and/or conduction problem of cardiac activity. It comprises a significant category of cardiovascular disorders that can occur alone or in conjunction with other cardiovascular diseases. Atrial fibrillation (AF) is one of the most frequent forms of human arrhythmias, with a significant disability and fatality rate in patients.^393,396,397^ The etiology of AF is linked to MMP-9; the increased activity of MMP-9 causes atrial fibrosis and induces AF.^398^ Another study demonstrated that HIF-1α stimulates the downstream factor TGF-β1 by promoting the expression of angiotensin II (Ang II), which causes high expression of MMP-9.^399^ Conversely, the levels of TGF-β1 and MMP-9 are lowered by inhibited HIF-1α expression, reducing the degree of atrial fibrosis.^399^ Ogi et al. reported a high HIF-1α level in AF patients. The study also postulated that the subsequent structural remodeling is caused by cardiac hypoxia.^400^ HIF-1 has been observed in peri-left atrial adipose and linked to fibrotic remodeling, which creates a substrate for AF.^401^ Xu et al. discovered that patients with permanent or persistent AF had higher levels of HIF-1α expression in the left atrial biopsies compared to patients with paroxysmal AF or patients in sinus rhythm from left atrial samples, implying a significant role of the protein in structural remodeling that supports AF initiation and propagation.^402^ Also, an increasing number of target genes have been discovered to play a role in various physiological and pathological processes in HIF-mediated AF.^403,404^

#### Hypoxia in congenital heart disease (CHD)

CHD is the most common type of congenital deformity, classified into three types based on hemodynamics: no shunt, left to right shunt, and right to left shunt.^405–407^ Patients with cyanotic CHD (CCHD) might have a hypoxic response, which leads to abnormalities in endothelial function, vascular remodeling, and thrombosis after emergency surgery.^408^ Prolyl-4-hydroxylase2 (PHDP2)/HIF-1α pathway is the key regulator under hypoxia. PHD2 activates HIF-1α oxygen-dependent hydroxylation of the internal oxygen-dependent degradation domain in a normoxic environment. However, this hydroxylation is inhibited during hypoxia, resulting in HIF-1α accumulation and vascular remodeling.^409^ Thus, it has been demonstrated that Egl-9 family hypoxia-inducible factor 1 (EGLN1) mutation decreases the hypoxic response of CCHD via the PHD2/HIF-1 pathway, which might be a viable target for CCHD therapy.^410^ Liu et al. discovered that Cited2 functional loss causes abnormalities in the heart and neural tube development, partially due to the regulation of HIF-1α transcriptional activity in the absence of Cited2,^411^ emphasizing its significant role in the development of CHD.

### Neurodegenerative diseases

Neurodegenerative disorders are characterized by the gradual death of susceptible groups of neurons; the frequency of this incidence increases rapidly with age.^412^ Three major neurodegenerative disorders are Alzheimer’s disease (AD), Parkinson’s disease (PD), and amyotrophic lateral sclerosis (ALS). Here, we discuss the function of hypoxia in neurodegenerative disorders.

AD is a serious neurodegenerative disease with a convoluted etiology and varying periods of onset, which is one of the most common neurodegenerative disorders.^413^ AD is distinguished by two key features: amyloid beta-peptide (Aβ) accumulation in the brain and the appearance of neurofibrillary tangles composed of hyperphosphorylated tau protein.^414^ Cerebral hypoxia is strongly related to AD, which is correlated to cardiovascular risk factors.^415^ Physical exercise lessens the incidence of AD, featured by functioning of the neurovascular unit.^416–418^ HIF-1α levels in the brain are lower in AD patients, which have been linked to increased phosphorylation of tau protein and production of neurofilament.^419^ Furthermore, the advancement of neurodegeneration is involved in an increase in the generation of ROS, contributing to decreased expression of genes essential for remaining nerve cell viability and synaptic transmission, especially the *HIF-1* gene.^414^

Another common age-related neurodegenerative disease is PD, which affects the elderly and is characterized by the loss of dopaminergic neurons and α-synuclein’s Lewy bodies (LB) accumulation.^420–423^ Accumulating evidence confirmed that mitochondrial malfunction and oxidative stress participate in the etiology of PD.^424^ Furthermore, HIF-1 is required for differentiation and survival of dopaminergic neuron, and a reduction in its expression results in neuronal death throughout the progression of PD.^425^ The in vitro and in vivo PD models revealed that the activation of HIF-1 exerts protective effects in neurons via expression of *EPO* and *VEGF* genes.^197,426,427^ Neuroprotective neuropeptide orexin-A induces HIF-1α expression, consequently activating VEGF and EPO in in-vitro PD models. Thus, HIF-1-mediated downstream signaling has the potential for PD treatment. In addition, the regulation of HIF-1 signaling by the ubiquitin-dependent proteasome pathway or HIF-specific prolyl hydroxylases is also able to avoid the neurons injury from oxidative stress, thereby accelerating the progress of PD.^401,428–430^

Amyotrophic lateral sclerosis (ALS) is a chronic neuronal disease caused by the injury to motor neurons in the motor cortex, spinal cord, and sub-brainstem.^431^ ALS causes gradual muscular weakening and atrophy of the muscles of the limbs, trunk, chest, and abdomen, which affects movement, communication, swallowing, and breathing, leading to death 3–4 years after the initial diagnosis.^432,433^ The dysregulation of EPO and VEGF accompanied by vascular changes, and blood flow disorder contributes to the pathogenesis of ALS, resulting in the hypoxia of the tissue.^434,435^ Hypoxia in tissues increases ROS production, leading to cell death.^436^ Thus, the uncontrolled hypoxia pathway is responsible for motor neuron death in ALS.^437^ Nomura et al. demonstrated that HIF-1α expression is dynamic in different stages of ALS, indicating the participation of HIF-1α in ALS.^438^ Dysregulation of the anti-hypoxic pathway induced by impaired HIF-1α activation promotes the motor neuron decline in ALS.^439,440^ Similar to the role in PD, HIF-1α activation protects the neurons in ALS. In an ALS in vivo model, the induction of HIF-1α decreases hypoxia-caused damage, protecting the neurons, reducing the inflammatory response, and lessening motor neuron degeneration.^438^ Conversely, decreased HIF-1α expression induced by ONO-1301-MS increases motor neuron generation in the mice model of ALS.^441^ Nonetheless, these findings need to be investigated further with respect to HIF-1α in ALS.

## Target therapeutics based on hypoxia

**Fig. 6:**
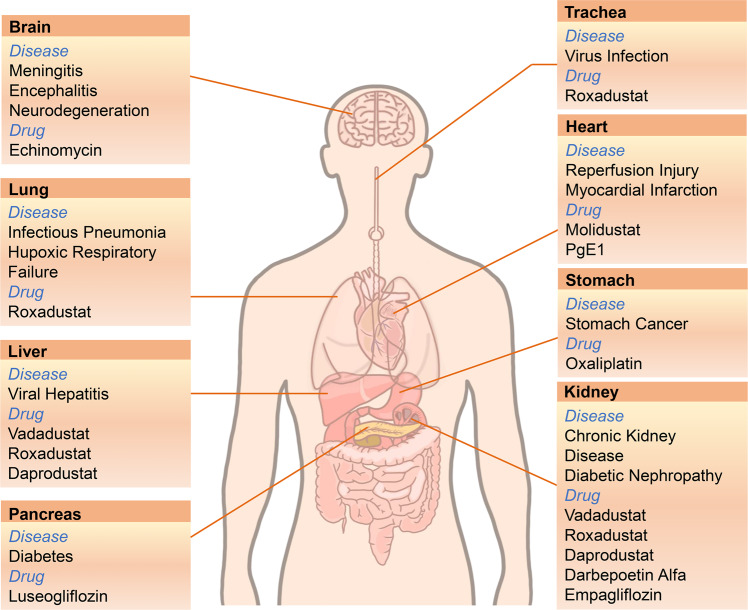
Developed drugs targeting hypoxia signaling in human diseases. The main human diseases in different organs are displayed with the according the developed drugs targeting hypoxia signaling

Oxygen balance ensures the normal progress of life activities. Hypoxia affects the expression of many genes with clinicopathological significance in various human diseases.^442^ HIF-1 is deemed as the core element in the hypoxia pathway. Based on the advance in human health and diseases involved in hypoxia, researchers have made a great effort to intervene in each step in the hypoxia signaling pathway upon the occurrence of diseases,^443^ to develop target therapeutics for hypoxia-associated diseases (Table 1). Next, we summarize the hypoxia-targeted therapeutics against major human diseases (Fig. 6).

**Table 1:** Summary of approved drugs in hypoxia-targeted therapeutics

Disease classification		Medicine name	Drug category	Stage	Typical example	Reference
Tumor		Belzutifan	HIF-2α specific antagonist	Approved by FDA	Renal cell carcinoma	^453–455^
		Oxaliplatin	DNA synthesis inhibitor	Approved by FDA	Colorectal cancer and liver cancer	^468,469^
Cardiovascular diseases		Molidustat	Prolyl hydroxylase inhibitor (PHI)	Approved by PMDA	CKD and diabetic heart	^487,488^
		Bosentan	Endothelin receptor antagonist	Approved by FDA	Raynaud syndrome	^491^
Metabolic diseases	Diabetes	Luseogliflozin	SGLT2 antagonist	Approved by PMDA	Diabetic nephropathy	^500^
	Chronic renal disease	Roxadustat	Prolyl hydroxylase inhibitor (PHI)	Approved by NMPA	Anemia in patients with CKD	^509–511^
		Daprodustat	Prolyl hydroxylase inhibitor (PHI)	Approved by MHLW	Anemia in patients with CKD	^513,514^
Infectious diseases	Respiratory infections	Roxadustat	Prolyl hydroxylase inhibitor (PHI)	Approved by NMPA	COVID-19	^515^

*FDA* the United States (U.S.) Food and Drug Administration, *PMDA* Pharmaceuticals and Medical Devices Agency of Japan, *NMPA* National Medical Products Administration of China, *MHLW* Ministry of Health, Labour and Welfare of Japan

### Hypoxia-targeted therapeutics in cancer and tumor

In the tumor hypoxic microenvironment, HIF functions in many aspects, such as improvement of glucose metabolism and enhancement of VEGF expression for angiogenesis to help the cells adapt to hypoxia. Abnormally high levels of angiogenesis, inflammation, and anaerobic glycolysis promote tumorigenesis and cause neoplastic diseases in the body.^444^ The stably generated HIF activates the downstream target genes successively, triggering a series of tumor activities. Therefore, HIF is considered one of the therapeutic targets of tumors.^445^ However, it may have varied roles in different tumor types. For example, the EGLN/HIF axis contributes to tumorigenesis in RCC,^446^ but has an opposite effect in other types of cancer.^447^ Thus, elucidating the exact role of HIFs in different conditions in the hypoxia-targeted therapeutics against tumors is recommended.

ccRCC is one of the common kidney cancers. The occurrence of pVHL tumor suppressor inactivation is a major event in ccRCC.^448^ Inactivation of pVHL stabilizes HIF-1α and HIF-2α. Therefore, several studies have focused on anti-caking agents for HIF-2α. PT2399 is a small-molecule inhibitor that dissociates HIF-2 and inhibits tumorigenesis in 56% of its congeners in human ccRCC cells.^449^ Compared to untreated controls, the growth of orthotopic tumors treated with PT2399 is arrested and regressed in mice.^450^ Another HIF2α-specific antagonist, PT2385, also inhibited the expression of HIF-2α target genes in ccRCC cell lines and mouse xenografts tumor model.^450^ PT2385 demonstrated a favorable safety profile in phase I dose-escalation trial and established the recommended phase II dose (RP2D) of 800 mg twice daily in humans.^451^ However, some analyses showed that patients are not benefitted clinically from PT2399.^452^ Belzutifan (MK-6482), a second-generation HIF2α anti-nodal agent, is efficacious in RCC and lung RCC in clinical trials and was subsequently approved for the treatment of VHL-associated diseases in August 2021.^453–455^ Topotecan, a HIF-1α inhibitor,^456^ exhibits antitumor activity in both in vivo and in vitro assays.^457^ Thus, it can be used for the treatment of multiple types of cancer, such as SCLC and ovarian cancer.^458,459^ The obvious decline in tumor blood flow and permeability was observed in 7/10 patients treated with topotecan over one treatment cycle.^460^.

Bortezomib (PS-341) is a proteasome inhibitor that inhibits HIF-1α activity by inhibiting the recruitment of P300 coactivators.^461^ A phase II trial showed that Bortezomib is ineffective in metastatic colon cancer but alters tumor response to hypoxia.^462^ The in vivo experiments of xenograft-bearing mice showed that bortezomib strongly inhibits VEGF production by up to 90%. This effect could be attributed to a decrease in HIF-1 transcriptional activity during treatment.^463^ RO7070179 is another HIF-1α inhibitor, shown in phase Ib clinical trial to reduce *HIF-1α* mRNA level in patients with hepatocellular carcinoma, thereby indicating its potential clinical benefit.^464^ Oxaliplatin, an antitumor drug, was used for the treatment of advanced CRC and GC.^465^ Several clinical trials have been conducted on oxaliplatin in combination with other drugs.^466–468^ Some studies indicated that the induction of HIF-1α degradation enhances the efficacy of oxaliplatin in CRC therapy.^469^ In addition, regulating the ubiquitination of HIF-1 is another strategy. Deubiquitinases (DUBs) can remove the ubiquitination of substrates, and the modulation of DUBs has now been identified as a promising drug target.^470^ *USP7*, one of the DUB genes, induces tumors by stabilizing HIF-1α.^471^ However, USP7 inhibitors slowed the tumor development in Lewis LC mice.^472^

In addition to the regulation of HIFs, applying the hypoxic properties of the tumor microenvironment to enhance the specificity of drugs is another therapeutic strategy. This class of drugs has minimal or no activity normoxically but can undergo bioreduction hypoxically to produce metabolites, known as hypoxia-activated prodrugs (HAPs), that are toxic to the cells.^473^ Evofosfamide (TH-302) is a HAP,^474^ which reduces tumor growth in neuroendocrine prostate cancer (NEPC).^475^ Multiple trials have investigated the antitumor efficacy of TH-302 in combination with other treatments. Data from a phase II trial in advanced pancreatic cancer patients showed that the combination of gemcitabine plus TH-302 significantly improves the progression-free survival (3.6 months in the gemcitabine group *vs*. 5.6 months in the combination group) and tumor response (3.6 months in the gemcitabine group *vs*. 5.6 months in the combination group).^476^ In a transgenic mouse model of adenocarcinoma, the combination of hypoxia-targeted therapy and checkpoint blockade controls tumor progression more significantly than either approach alone.^477^ The clinical data from another phase II trial of joint use of TH-302 and doxorubicin in advanced soft tissue sarcoma indicated that the combination therapy was superior to other first-line treatments, and TH-302 did not exhibit any hepatic, renal, or cardiac toxicity.^478^ Nonetheless, phase III data showed that compared to doxorubicin alone, the addition of TH-302 failed to improve the overall survival.^479^

The ErbB receptor tyrosine kinase family members are considered oncogenes in various cancers.^480^ Tarloxotinib is also a HAP that effectuates by inhibiting the activation of four members of the ErbB family. Also, it inhibits signaling and cell proliferation in patient-derived cancer cells in vitro and tumor growth in multiple mouse patient-derived xenograft models.^481^ Importantly, compared to 190 μmol/h/kg to the skin, the total tumor exposure to the metabolite tarloxotinib was 595 μmol/h/kg, indicating the specificity of this drug targeting tumor tissue. However, cancer patients receiving EGFR-targeted HAP therapy eventually develop drug resistance, including pancreatic or metastatic LC.^482^ Thus, these issues on drug resistance require further exploration.

### Hypoxia-targeted therapeutics in cardiovascular diseases

Stabilization of HIF-1α is a prerequisite for normal cardiac development.^483^ During the disease process, the expression of HIF-1α may be disturbed or inhibited, thereby triggering cardiac dysfunction.^484^ Ischemic preconditioning and reperfusion are common cardioprotective strategies.^485^ Also, the modulation of HIF-1α expression with drugs is one of the therapeutic directions, facilitating hydroxylate of HIF-1α and ubiquitin-dependent degradation.^486^

Molidustat stabilizes HIF-1α and its downstream target genes in T2D cardiomyocytes. In T2D rats, oral administration of molidustat increases the body’s HIF targets and improves the recovery of ischemia-reperfusion by 27%.^487^ It also reduces fatty acid metabolism in the heart, which is shown as a 70% reduction in myocardial triglycerides.^487^ Several studies have assessed molidustat for the therapy of chronic kidney disease and anemia.^488–490^ Thus, its potential in the treatment of cardiovascular diseases may be investigated in future studies.

Raynaud’s syndrome is characterized by vasospasm that restricts blood flow leading to hypoxia, with markedly elevated levels of HIF-1α in both monocytes and serum. The combination of prostaglandin E1 (PgE1) and the endothelin-1 blocker bosentan can prevent its increase but not PgE1 administration alone.^491^ Data from previous studies suggested that PgE1 stimulates neovascularization by upregulating VEGF in patients with ischemic heart disease.^492^. PgE1 is a pulmonary vasodilator that needs to be evaluated in neonatal hypoxic respiratory failure.^493^

In addition to removing factors that interfere with HIF-1α expression, exogenous administration of HIF-1α may also achieve therapeutic purposes. A study showed that exosomes (Exo) modified with HIF-1α enhance the proliferation of human umbilical vein endothelial cells injured by hypoxia preconditioning.^494^. Exo-HIF-1α significantly reduced left ventricular fibrosis area ratio and inner peripheral fibrosis length compared to the Exo group with upregulated pro-angiogenic factors.

### Hypoxia-targeted therapeutics in metabolic diseases

#### Diabetes

HIF-1α plays a vital role in metabolic diseases in tissues or organs.^149^ Diabetes is one of the most common metabolic diseases, and 90–95% of adults with diabetes worldwide have T2D.^495^ The regulation of HIF-1α in β-cell reserve and aryl hydrocarbon receptor nuclear translocator expression in islets. When HIF-1α in β cells was disrupted, mice exhibited glucose intolerance and β-cell abnormality; these conditions were improved when HIF-1α levels were restored, suggesting that HIF-1α is a T2D β-cell potential therapeutic target for functional disorders.^496^ Li et al. reported a HIF-1α stabilizer 1a that induces the activation and accumulation of HIF-1α and its driving genes in a diabetic mouse model.^497^ Intrarenal hypoxia is detected in diabetic patients, and HIF-1 regulates the occurrence of tubulointerstitial fibrosis. Sodium-glucose cotransporter 2 (SGLT2) inhibitor protects the kidney by inhibiting HIF-1α expression.^498^ Luseogliflozin, an SGLT2 inhibitor, relieves renal tubular damage and interstitial fibronectin in diabetic mice by inhibiting HIF-1α accumulation that reduces mitochondrial oxygen consumption.^499^ The treatment with luseogliflozin in mice with inhibited insulin and IGF-1 target receptors showed improved β-cell proliferation and hyperglycemia, but not hyperinsulinemia.^500^ Empagliflozin is a highly selective SGLT2 inhibitor and well-tolerated in humans.^501,502^ In T2D patients, the addition of empagliflozin to the standard of care reduces the progression of kidney disease compared to placebo (12.7% of the empagliflozin group *vs*. 18.8% of the placebo group). Strikingly, renal replacement therapy was initiated in 0.3% of patients receiving empagliflozin, compared to twice as high in the control group.^503^ Notably, the oxidative stress involved in insulin resistance needs to be considered.^504^

#### Chronic kidney disease (CKD)

Erythropoiesis-stimulating agents (ESAs) and prolyl hydroxylase inhibitors (PHIs) are commonly used to treat CKD. However, statistical analysis demonstrated that long-term ESA use might increase the risk of death.^505^ Therefore, lower doses should be used whenever possible in CKD patients with cancer receiving ESA.^506^ Unlike ESA requiring injection, PHI is a class of oral medications that reduce the cost and risks of treatment for patients.^507^ Well-studied PHIs contain vadadustat, roxadustat, and daprodustat. PHIs stabilize HIF and stimulate EPO and erythropoiesis. In a phase III trial, vardarestat was compared to darbepoetin alfa in ESA. The pooled analysis showed that the hazard ratio for major adverse cardiovascular events was 1.17, which did not meet the prespecified non-inferiority of 1.25 but achieved the prespecified non-inferiority for hematologic efficacy.^508^ Roxadustat has been authorized for China in dialysis-dependent CKD anemia patients’ treatment. A phase II trial showed hemoglobin levels increased by 1.9 ± 1.2 g/dL in patients with CKD in the roxadustat group compared to the baseline mean and a slight decrease in the placebo group.^509^ The level of total cholesterol was lower in the roxadustat group than that in the placebo group.^510^ However, patients receiving roxadustat were likely to develop hyperkalemia or metabolic acidosis. A phase III trial in CKD patients with anemia showed that roxadustat had a slightly higher (almost the same) incidence of adverse events than the placebo group, whereas roxadustat significantly reduced the risk of red blood cell transfusion.^511^ Another phase III trial showed that roxadustat was non-inferior to darbepoetin alfa in maintaining hemoglobin.^512^ Daprodustat was also non-inferior to darbepoetin alfa in terms of hazard ratios for adverse events and maintenance of hemoglobin levels in anemic patients with or without dialysis.^513,514^ In the above events, the data from clinical trials of PHIs display the comparative efficacy of ESA.

### Hypoxia-targeted therapeutics in infectious diseases

#### Respiratory system infection

In the most common infectious respiratory diseases caused by influenza virus and coronavirus infection, oxygen tension is considered a non-negligible factor in viral replication.^230^ As mentioned, HIF-1α facilitates SARS-CoV-2 replication and amplifies inflammatory response,^146,147^ suggesting that HIF regulation is a promising therapeutic target. However, the roles of HIF vary at different stages of the viral infection in COVID-19 patients. The evidence has shown that the SARS-CoV-2 receptor ACE2 can be reduced by roxadustat through a HIF-1α-dependent pathway, which inhibits virus entry and replication.^515^ HIF-1α, on the other side, can boost the activity of Cathepsin L which can cleave S protein. Early use of PHD may aid viral replication.^516^ And it may also participate in the cytokine storm generated by SARS-CoV-2 through its stimulating influence on the expression of macrophage migration inhibitory factor (MIF).^517^ It is reported that dexamethasone can break the link between HIF and MIF.^518^ The expression of HIF-1α associated with macrophage inflammation in COVID-19 patients is elevated.^519^ Upon viral infection, SARS-CoV-2 damages the mitochondria and triggers ROS production, thereby inducing HIF-1α, promoting viral replication, and aggravating the inflammatory response.^146,147^ In conclusion, HIF-1α may have opposite effects on various aspects of virus invasion activities, and it is necessary to carefully evaluate the measures that need or can be taken according to the conditions of patients.

The studies in influenza A virus (IAV)-infected mice showed that after knockout of HIF-1α in lung epithelial cells, the mice exhibited severe lung inflammation.^520^ Tissue macrophages produce inflammatory mediators during pathogen infection, which is regulated by β-catenin-HIF-1α signaling, and Wnt promotes the interaction between these two signaling molecules. Data from a mouse model of influenza virus pneumonia showed that β-catenin-mediated inflammation in macrophages increases acute host morbidity.^519^ Therefore, the role of HIF-1α in different tissues should be reconsidered when targeting HIF-1α therapeutically.

#### Digestive system infection

Hypoxic environment is not conducive to virus replication, but studies have found that HBV can use hypoxia signaling pathway to generate in hypoxic environment,^521^ Chronic stabilization of HIF exhibits deleterious effects on the body.^522^ As mentioned above, in liver cancer cells, the activity of HBV enh1 is enhanced.^223^ Therefore, in addition to using HIF inhibitors to reduce the expression of HIF, this specific activity can also be used to construct a specific expression system for targeted gene therapy.

*Helicobacter pylori* (*H. pylori*) is associated with a large number of gastrointestinal diseases,^523^ and is known as one of the leading factors affecting the development of GC.^524,525^ Therefore, the treatment of *H. pylori* is crucial for preventing GC. *H. pylori* infection may trigger duodenal ulcers, a type of peptic ulcer that is more common than gastric ulcers. Some studies demonstrated that in the process of duodenal ulcer, ischemia induces HIF-1α expression and angiogenesis factors production including VEGF.^526^ Clinical trials demonstrated *H. pylori* eradication for the treatment of *H. pylori*-associated duodenal ulcers.^527^ Reportedly, *H. pylori* infection increases the expression of HIF-1α.^528^ Consequently, the hypoxia signaling pathway may be one of the targets of treatment and illuminates the research on the treatment of other diseases caused by *H. pylori*. Therapies targeting the hypoxic pathway may be useful in the treatment of pathogens infections of the digestive system.

#### Nervous system infection

Hypoxia takes part in the pathogenesis of many neurological diseases.^235^ Meningitis, encephalitis, and even Alzheimer’s disease is a group of diseases caused by infection or autoimmunity.^529^ Among most cases of viral infection, enterovirus is the main agent,^530^ and mumps, lymphocytic choriomeningitis, and type I and II scab viruses are also common pathogens.^531^ Enterovirus 71 (EV71) is a common enterovirus that causes neurological diseases in severe cases, and hypoxia may be one of the participants in the neuropathogenesis of EV71.^532^ In a consistent study, the constructed immunocompetent or immunodeficient mouse models have white plaques in the muscles after infection with EV71, which are related to hypoxia.^533^ Progressive multifocal leukoencephalopathy (PML) is a kind of organic brain disease caused by Polyomavirus JC (JCV). HIF-1α activates the JCV virus promoter, implying a cure for the occurrence of PML.^534^

Furthermore, neurological diseases are also caused by bacterial infection. For example, *S. pneumoniae* infection can cause fatal bacterial meningitis.^535^ HIF-1α inhibitor echinomycin can improve blood-brain barrier function and increase the survival in *S. pneumoniae*-infected mice.^536^ In neuroinfection events, the investigation of the role of HIF-1α might help to understand the neuropathogenesis and develop treatment options.

## Prospects in therapeutics of hypoxia-associated diseases

**Fig. 7:**
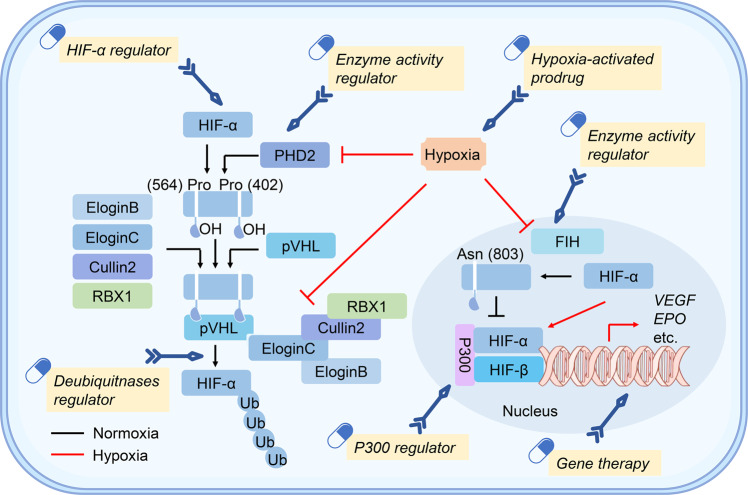
The principle of therapeutics targeting hypoxia signaling. The stratigies of therapeutics targeting hypoxia signaling are classified in (1) HIF-1α regulator; (2) Enzyme activity regulator; (3) deubiquinases regulator; (4) hypoxia-activated prodrug; and (5) P300 regulator

Hypoxia signaling participates in events of cellular viability and activity to respond to oxygen deprivation. HIF-1 is the central regulator modulated from upstream signals or stimuli and induces downstream gene transcription, which has been implicated in several human diseases. Owing to its control of various diseases, HIF-1 (mainly HIF-1α and HIF-1β) is preferred in the development of targeted therapy. Several strategies are available for therapeutics against hypoxia-associated diseases (Fig. 7). (1) Alteration of HIF-1 transcription by the upstream signals or stimuli; (2) Regulation of HIF-1 stability via interfering protein modification, such as deSUMOylation and deubiquitination; (3) Control of HIF-1 function by disturbing related enzyme activity in the complex. The above strategies are attributed to HIF intervention either directly (the expression and activity) or indirectly (co-activator and repressor). Therefore, the scope of drug screening or repurposing in the development of therapeutics against hypoxia-associated diseases has been clarified.

Hypoxia is the status of the microenvironment in the body.^537^ HIF-1 regulates various target genes in corresponding diseases. Typically, the hypoxia-targeted therapies are discrepant and may also have opposite effects in the treatment of various diseases. As a result, the effect of hypoxia-targeted therapy interventions on spatiotemporal behaviors in diseases is yet to be investigated. One possible way to improve the efficiency of hypoxia-targeted therapy could be the combination of specific drugs against the diseases. The advantages of this approach are improving the drug effects and eliminating drug resistance. Another aspect may be the modification of the drug and the design of the delivery system, thereby increasing effective hypoxia-targeted therapies. For example, PEGylated biopharmaceuticals are used to improve the physicochemical properties and biological responses of a drug. The use of exosomes as the drug-delivery system would reduce immunogenicity as the therapeutic tool for hypoxia-associated diseases.

The tissue- or disease-specificity of targeted therapy must be considered since improper regulation of HIF or its downstream genes in normal tissues may have harmful consequences in cells or tissues. Agent-targeted therapy does not appear to be very selective, as the same drug can affect multiple organs. Roxadustat, for example, is primarily used to treat anemia in individuals with renal illness, but it has also been proven to affect hepatic lipolysis.^538,539^ As a result, the danger that other non-diseased tissues may carry during administration should be thoroughly assessed. Even in the same tissue, HIF may play conflicting roles in various disease processes. Stable HIF expression, for example, protects against acute lung injury during hypoxia and promotes pulmonary hypertension development.^522^ The hypoxic prodrug, on the other hand, has a somewhat higher specificity because its active form requires a certain oxygen concentration to activate. Gene therapy can be also highly tissue-specific. Hypoxia-specific expression system^540^ constructs an oxygen concentration-dependent gene expression vector^541^ by inserting the hypoxia response element HRE from different hypoxia-inducible genes into the upstream of the SV40 minimal promoter. This is especially important for solid tumors.

The hypoxic environment is a factor impacting the efficiency of several tumor treatment techniques, yet this characteristic environment is currently being utilized for tumor-targeted therapy. The plasmid will be highly expressed selectively in specific hypoxic locations in this way. Leaky expression is a serious issue that requires immediate care.^542^ To improve tissue specificity, a promoter that is active exclusively in a certain tissue or place, such as human tumor cells, can be added to the expression system. For instance, the survivin promoter is the sole one,^87,543^ which could increase the target selectivity of some HIF-1 oxygen-independent cancer therapies.

Among several HIFs, HIF-1α is the primary option to develop target drugs in hypoxia-associated diseases. Most developed drugs in clinical trials are designed on the basis of the direct and indirect regulation of HIF-1α (Table 2). Fortunately, emerging agents targeting HIF-2α are promising anti-tumor therapeutics, providing alternative candidates for hypoxia-targeted drugs when all HIFs beyond HIF-1α are taken into consideration. Finally, with the concerted help of updated basic research on hypoxia-related diseases and advances in multidisciplinary fields, such as structural biology, medicine, chemistry, and pharmacy, therapeutics against hypoxia-associated diseases have novel avenues.

**Table 2:** Clinical trials of develped drugs in hypoxia-targeted therapeutics

Disease classification		Medicine name	Drug category	Phase	NCT Number	Time		Locations
						First Posted	Last Update Posted	
Tumor		Topotecan	HIF-1α antagonist	Phase 1 Phase 2	NCT00005793	2003-05-07	2012-09-25	H. Lee Moffitt Cancer Center and Research Institute Tampa, FL, U.S.
				Phase 1	NCT00765973	2008-10-03	2020-11-13	Barbara Ann Karmanos Cancer Center Detroit, MI, U.S.; South Texas Accelerated Research Therapeutics San Antonio, TX, U.S.
				Phase 2	NCT00601003	2008-01-25	2022-04-28	Rady Children’s Hospital San Diego, California, United States; Connecticut Children’s Hospital Hartford, CT, U.S.; Arnold Palmer Hospital for Children- MD Anderson Orlando, FL, U.S.
				Phase 1	NCT01670175	2012-08-22	2017-06-21	UCSF Benioff Children’s Hospital San Francisco, CA, U.S.
				Phase 2	NCT01931098	2013-08-29	2020-11-24	National Institutes of Health Clinical Center, 9000 Rockville Pike Bethesda, MD, U.S.
				Phase 1 Phase 2	NCT02100007	2014-03-31	2017-10-02	Pinnacle Oncology Hematology Scottsdale, AZ, U.S.; University of Colorado Cancer Center Aurora, CO, U.S.; Northwestern University Chicago, IL, U.S.
				Phase 1 Phase 2	NCT02487095	2015-07-01	2022-04-12	National Institutes of Health Clinical Center, 9000 Rockville Pike Bethesda, MD, U.S.
				Phase 1	NCT04047251	2019-08-06	2022-04-19	HonorHealth Scottsdale, Arizona, United States; Sarah Cannon Research Institute at HealthONE Denver, CO, U.S.; Dana Farber Cancer Institute (DFCI) Boston, MA, U.S.
				Phase 1 Phase 2	NCT02866006	2019-08-06	2022-04-19	Samsung Medical Center Seoul, Korea, Republic of
				Phase 3	NCT04799002	2021-03-16	2021-03-16	Sun Yat-sen University Guangzhou, Guangdong, China
		Bortezomib	Proteasome inhibitor	Phase 1 Phase 2	NCT01522872	2012-02-01	2016-06-02	Pacific Cancer Care Monterey, CA, U.S.; Moffitt Cancer Center Tampa, Florida, United States; Maine Center for Cancer Medicine Scarborough, ME, U.S.
		RO7070179	HIF-1α antagonist	Phase 1	NCT02564614	2015-10-01	2018-02-15	Indiana University Indianapolis, IN, U.S.; Laura and ISAAC Perlmutter Cancer Center at NYU Langone. New York city, NY, U.S.; NYU Langone Medical Center; Bellevue Hospital New York city, NY, U.S.
		Evofosfamide	Small molecule inhibitor	Phase 1	NCT00495144	2007-07-02	2012-07-27	TGen Drug Development Services Scottsdale, AZ, U.S.; Mayo Clinic Arizona Scottsdale, AZ, U.S.; St. Mary’s Medical Center San Francisco, CA, U.S.
				Phase 1 Phase 2	NCT00743379	2008-08-28	2015-05-07	Mayo Clinic Cancer Center Scottsdale, AZ, U.S.; Premiere Oncology of Arizona Scottsdale, AZ, U.S.; Indiana University Cancer Center Indianapolis, IN, U.S.
				Phase 1	NCT01149915	2010-06-24	2015-05-07	University of Texas M.D. Anderson Cancer Center Houston, TX, U.S.
				Phase 1	NCT01497444	2011-12-22	2020-02-06	Mayo Clinic Scottsdale Scottsdale, AZ, U.S.; Mayo Clinic Cancer Center Rochester, MN, U.S.
				Phase 1 Phase 2	NCT01522872	2012-02-01	2016-06-02	Pacific Cancer Care Monterey, CA, U.S; Moffitt Cancer Center Tampa, FL, U.S.; Maine Center for Cancer Medicine Scarborough, MW, U.S.
				Phase 1	NCT03098160	2017-03-31	2017-10-30	MD Anderson Cancer Center Houston, TX, U.S.
		Th-302 combined with Gemcitabine	Small molecule inhibitor	Phase 1 Phase 2	NCT00743379	2008-08-28	2015-05-07	Mayo Clinic Cancer Center Scottsdale, AZ, U.S.; Premiere Oncology of Arizona Scottsdale, AZ, U.S.; Indiana University Cancer Center Indianapolis, IN, U.S.
		Tarloxotinib	HER kinase inhibitor	Phase 2	NCT02454842	2015-05-27	2017-02-27	University of Southern California-Norris Los Angeles, CA, U.S.; St. Joseph Heritage Healthcare Santa Rosa, CA, U.S.; University of Colorado Cancer Center Aurora, CO, U.S.
				Phase 2	NCT02449681	2015-05-20	2017-02-27	University of Southern California-Norris Los Angeles, CA, U.S.; Stanford school of Medicine Stanford, CA, U.S.; Georgetown Medical Center Washington, DC, U.S.
Metabolic diseases	Diabetes	Empagliflozin	SGLT2 antagonist	Phase 4	NCT02932436	2016-10-13	2021-04-19	Universitätsmedizin der Johannes Gutenberg-Universität Mainz, Zentrum für Kardiologie, Präventive Kardiologie und Medizinische Prävention Mainz, Germany
				Phase 2	NCT03078101	2017-03-13	2019-08-08	Department of Internal Medicine III, Division of Nephrology and Dialysis, Medical University of Vienna, Austria Vienna, Austria
				Phase 1	NCT03895229	2019-03-29	2019-04-02	Drug research centre Cairo, Egypt
				Early Phase 1	NCT04203927	2019-12-18	2022-02-10	University of Virginia Charlottesville, VA, U.S.
				Phase 2	NCT04662866	2020-12-10	2021-04-08	Oslo University Hospital, Aker Hospital Oslo, Norway
				Phase 3	NCT05139472	2021-12-01	2021-12-01	Institute for Exercise and Environmental Medicine Dallas, TX, U.S.; University of Texas Southwestern Medical Center Dallas, TX, U.S.
				Phase 2	NCT05174507	2021-12-30	2022-04-20	Department of Endocrinology, Diabetes and Metabolism, University Hospital Basel Basel, Switzerland
				Phase 4	NCT05210517	2022-02-27	2022-02-27	VU University Medical Center Amsterdam, Noord-Holland, Netherlands
	Chronic renal disease	Vadadustat	Prolyl hydroxylase inhibitor (PHI)	Phase 1	NCT02412449	2015-04-09	2018-11-14	Kalamazoo, MI, U.S.
				Phase 3	NCT02680574	2016-02-11	2021-06-22	Research Sites Birmingham, Huntsville, and Tuscumbia, AL, U.S.
				Phase 3	NCT02865850	2016-08-15	2021-02-02	Research Site Huntsville, AL, U.S.; Research Site Mesa, AZ, U.S.; Research Site Anaheim, CA, U.S.
				Phase 3	NCT02892149	2016-09-08	2021-02-26	Research Site Huntsville, AL, U.S.; Research Site Mesa, AZ, U.S.; Research Site Pine Bluff, AR, U.S.
				Phase 2	NCT03054350	2017-02-15	2021-04-08	Aichi, Japan; Ehime, Japan; Fukui, Japan
				Phase 2	NCT03140722	2017-05-04	2021-02-21	Research Sites Bakersfield, Elk Grove, and Encino, CA, U.S.
				Phase 3	NCT03242967	2017-08-08	2018-11-05	Research Site Northridge, CA, U.S.
				Phase 1	NCT03639155	2018-08-21	2019-03-22	Research Site Baltimore, MD, U.S.

NCT number, The National Clinical Trial number is generated in ClinicalTrials.gov when the assigned study is registered. The information of clinical trails is avaiable from https://clinicaltrials.gov. The date is expressed as year-month-day
